# Acute and chronic blood serum proteome changes in patients with methanol poisoning

**DOI:** 10.1038/s41598-022-25492-9

**Published:** 2022-12-09

**Authors:** Pavel Cejnar, Tatiana Anatolievna Smirnova, Stepanka Kuckova, Ales Prochazka, Ivan Zak, Karel Harant, Sergey Zakharov

**Affiliations:** 1 Department of Computing and Control Engineering, University of Chemistry and Technology, Prague, Technicka 5, 166 28 Prague 6, Czech Republic; 2 University Hospital Hradec Kralove, Sokolska 581, 500 05 Hradec Kralove, Czech Republic; 3 Department of Biochemistry and Microbiology, University of Chemistry and Technology, Prague, Technicka 5, 166 28 Prague 6, Czech Republic; 4 Department of Occupational Medicine, First Faculty of Medicine, Charles University, Na Bojisti 1, 12000 Prague, Czech Republic; 5 Toxicological Information Centre, General University Hospital, Na Bojisti 1, 120 00 Prague 2, Czech Republic; 6 Proteomics Core Facility, Faculty of Science, BIOCEV, Charles University, Prumyslova 595, 252 42 Vestec, Czech Republic

**Keywords:** Diagnostic markers, Proteomics

## Abstract

Twenty-four blood serum samples from patients with acute methanol poisoning (M) from the mass methanol poisoning outbreak in the Czech Republic in 2012 were compared with 46 patient samples taken four years after poisoning (S) (overlap of 10 people with group M) and with a control group (C) of 24 samples of patients with a similar proportion of chronic alcohol abuse. When comparing any two groups, tens to hundreds of proteins with a significant change in concentration were identified. Fifteen proteins showed significant changes when compared between any two groups. The group with acute methanol poisoning showed significant changes in protein concentrations for at least 64 proteins compared to the other groups. Among the most important identified proteins closely related to intoxication are mainly those involved in blood coagulation, metabolism of vitamin A (increased retinol-binding protein), immune response (e.g., increased complement factor I, complement factors C3 and C5), and lipid transport (increased apolipoprotein A I, apolipoprotein A II, adiponectin). For blood coagulation, the most affected proteins with significant changes in the methanol poisoning group were von Willebrand factor, carboxypeptidase N, alpha-2-antiplasmin (all increased), inter-alpha-trypsin inhibitor heavy chain H4, kininogen-1, plasma serine protease inhibitor, plasminogen (all decreased). However, heparin administration used for the methanol poisoning group could have interfered with some of the changes in their concentrations. Data are available via ProteomeXchange with the identifier PXD035726.

## Introduction

Methanol is widely used in industry, agriculture, and customer services throughout the world as a solvent, freezing agent, a component of windshield washer fluids, fuel, and for chemical synthesis of other substances^1–3^. Cases of accidental or deliberate acute exposure are reported frequently. Mass methanol poisoning outbreaks due to the consumption of adulterated alcoholic beverages present a serious challenge because of the high mortality rate and long-term or permanent central nervous system and visual damage in survivors^4–7^.

After absorption, about 90% of methanol is rapidly oxidized by alcohol dehydrogenase (ADH), catalase, and cytochrome P2E1 of the microsomal oxidizing system to formaldehyde and by aldehyde dehydrogenase to formic acid, which inhibits the mitochondrial respiratory chain by blocking cytochrome c oxidase, resulting in ATP depletion, lactate accumulation, and severe metabolic acidosis^8,9^.

Methanol-induced brain basal ganglia damage, mostly bilateral necrosis of the putamen, and lesions in subcortical white matter are typical magnetic resonance findings associated with severe neurological sequelae (secondary parkinsonism) in survivors^10,11^.

Neuroinflammation, a natural response of the brain immune system to both traumatic and non-traumatic injury, results in the release of many proinflammatory mediators, including leukotrienes and cytokines^12–14^. These mediators are also involved in the development of brain edema and disruption of the blood–brain barrier. Some of these mediators can reach the peripheral circulation through disruption of the blood–brain barrier and can also be measured in the blood serum^15^. Higher concentrations of inflammatory mediators can also be measured in methanol-poisoned patients as a response to either brain lesions or disruption of the blood–brain barrier^12^. The complement system has homeostatic functions in the uninjured central nervous system, but after any traumatic brain injury, it is also thought to be responsible for initiating and amplifying neuroinflammation in the stressed and injured central nervous system^16–18^.

Clinical symptoms of methanol-induced optic neuropathy range from blurred vision, decreased visual acuity, photophobia, and altered visual fields to complete blindness. The retinal ganglion cells and their axons, which form the optic nerve, are selectively vulnerable to histotoxic hypoxia caused by formic acid^19,20^. The inhibition of the mitochondrial oxidative phosphorylation process through the binding of formic acid with cytochrome c oxidase is the main mechanism of methanol-induced optic neuropathy; however, other mechanisms, including intensification of the oxidative peroxidation process with the formation of cytotoxic compounds, as well as an increase in the synthesis of proinflammatory cytokines, and influence on the expression of key proteins responsible for maintaining cell homeostasis, also play an important role^12,21,22^.

Methanol toxic intermediate metabolite formaldehyde reacts with glutathione and amino acid residues^1^, which leads to the alteration of the structure and biological activity of peptides and proteins, and also increases the sensitivity of altered proteins to proteases^23^.

Mass methanol poisoning outbreak with more than 130 cases and more than 50 deaths that occurred in the Czech Republic^7,24^ from September to December of 2012 led to more than a hundred hospitalized patients. Patients with acute methanol poisoning had two major groups of clinical manifestations (if they survived the poisoning): (a) brain lesions (52% of survived patients), either hemorrhagic (33%) or non-hemorrhagic (19%), most frequently in the basal ganglia of the brain, of which especially in the putamen^25^; (b) damage of retina and axons of the optic nerve, which was manifested by visual impairment of varying degrees (31%) and later chronic neurodegeneration of the retina (24%)^26^.

Proteomic analysis of biological matrices, such as blood serum, urine, and exhaled breath condensate, is an important research tool in human and clinical toxicology that brings new data^27,28^. Serum proteomics can reflect systemic and target organ impairments and confirm specific modifications to selected proteins. The formaldehyde-induced alterations in proteins, histotoxic hypoxia caused by formic acid, and damage of the blood**–**brain barrier, brain structures, optic nerve, retina, and blood**–**retinal barrier can lead to the serum proteome changes relevant to the prognosis and clinical management of methanol intoxication. Thus a longitudinal, observational cohort study was carried out on patients’ blood serum samples to reveal changes in proteome regarding (a) neuronal-function related or neuroimmune and inflammatory response proteins, which could originate from damaged brain structures and be released into the blood through a broken blood–brain barrier; (b) retinal-function related proteins, which could originate from the damaged retina and can also be released through the blood**–**retinal barrier; and (c) coagulation related proteins, which could originate from hemorrhagic brain lesions and coagulation disorders. Furthermore, the proteomic analysis may also provide important information for the timely identification of long-term health sequelae of poisoning. Therefore, our aim was also to analyze the human blood serum proteomic profile in patients hospitalized with acute methanol poisoning and compare them to blood serum proteomic profiles of survivors several years after the exposure.

## Materials and methods

### Study design and setting

A prospective, longitudinal, single-center, observational cohort study was carried out on patients treated in hospitals with confirmed acute methanol poisoning. A total of 108 patients [mean age with standard deviation (SD) of 50.9 ± 2.6 years] were treated in the hospital. 84 patients with a mean age of 49.9 ± 3.0 years survived and were discharged. Of those who survived the poisoning, 55 patients aged 46.7 ± 3.6 years (84% males, 16% females) provided their informed consent and were recruited into the prospective study of long-term health sequelae of poisoning^29^. Of these patients, 37 (67%) had a history of chronic alcohol abuse. Clinical and laboratory tests indicating acute exposure were collected using standardized data collection protocols. Information regarding treatment modalities used and outcomes was collected from discharge reports.

### Blood serum samples

Venous blood for proteomic analysis was obtained from 24 patients with confirmed acute methanol poisoning upon admission to the hospital (group M (“Methanol”) in the study) with heparin administration for hemodialysis and ethanol or fomepizole administration as the antidote to block ADH^30^. Blood samples were spun, the serum was separated, and the samples were frozen at − 80 °C until the analyses. In the follow-up group of survivors of methanol poisoning (group S (“Survivors”) in the study), venous blood samples for proteomic analysis were obtained from 46 patients during the examination, which took place 4 years after discharge from the hospital. In this group, for 10 patients (subgroup S_pair_ within group S), the corresponding acute venous samples drawn during hospitalization were available (subgroup M_pair_ within group M). For the control group not exposed to methanol, 24 healthy subjects with an age of 44.0 ± 4.2 years and with the same ethnicity and a similar history of chronic alcohol abuse (63%) were recruited (group C, “Controls”). The data from groups S and C were also combined (referred to as group SC) where suitable to compare the acute proteomic profile (group M) with the non-acute proteomic profile.

### Sample preparation and LC–MS/MS analyses

#### Serum depletion

For depletion of the 14 most abundant serum proteins, an Agilent MARS 14 column pn: 5188–6557 (Agilent) with buffers pn: 5185–5987, pn: 5185–5988 and spin columns pn: 5185–5990 was used (all Agilent). 20 µL of serum was loaded onto the column. Depletion of samples was done on a DIONEX Ultimate 3000 HPLC (Thermo Fisher Scientific) with UV detection of eluted proteins. Fractions were collected with an in-house manufactured fraction collector. Four fractions (400 µL) containing the proteins of interest were precipitated with 4 volumes of cold acetone and resuspended in 100 mM triethylammonium bicarbonate (TEAB, Thermo Fisher Scientific, pn: 90,114).

#### Protein digestion

Proteins from the depleted serum in 100 mM TEAB with 2% sodium deoxycholate (SDC, Sigma-Aldrich) were boiled at 95 °C for 5 min. The protein concentration was determined using a BCA protein assay kit (Thermo Fisher Scientific), and 20 µg of protein per sample was used for the mass spectrometry (MS) sample preparation. Cysteines were reduced with a 10 mM final concentration of tris(2-carboxyethyl)phosphine hydrochloride (TCEP, Sigma-Aldrich) and blocked with a 40 mM final concentration of chloracetamide (60 °C for 30 min). Samples were digested with trypsin (trypsin/protein ratio of 1/30) at 37 °C overnight. After digestion, samples were acidified with trifluoroacetic acid (TFA, Sigma-Aldrich) to 1% final concentration. SDC was removed by extraction to ethylacetate^31^, and peptides were desalted using in-house-made stage tips packed with C18 disks (Empore) according to^32^.

#### nLC-MS/MS analysis

Nano reversed-phase columns (EASY-Spray column, 50 cm × 75 µm ID, PepMap C18, 2 µm particles, 100 Å pore size, Thermo Fisher Scientific) were used for nano-liquid chromatography-tandem mass spectrometry (nLC-MS/MS) analysis. Mobile phase buffer A was composed of water and 0.1% formic acid. Mobile phase B was composed of acetonitrile and 0.1% formic acid. Samples were loaded onto the trap column (C18 PepMap100, 5 μm particle size, 300 μm × 5 mm, Thermo Fisher Scientific) for 4 min at 18 μL/min. The loading buffer was composed of water, 2% acetonitrile, and 0.1% trifluoroacetic acid. Peptides were eluted with mobile phase B gradient from 4 to 35% B in 60 min. Eluting peptide cations were converted to gas-phase ions by electrospray ionization and analyzed on a Thermo Orbitrap Fusion (Q-OT-qIT, Thermo Fisher Scientific). Survey scans of peptide precursors from 350 to 1400 m/z were performed using the orbitrap at 120 K resolution (at 200 m/z). Tandem MS was performed by isolation at 1.5 Th with the quadrupole, higher energy collisional dissociation (HCD) fragmentation with a normalized collision energy of 30, and rapid scan MS analysis in the ion trap. The MS2 ion count target was set to 104, and the max injection time was 35 ms^33^. The acquired mass spectrometry proteomics data were deposited to the ProteomeXchange Consortium via the PRIDE^34^ partner repository with the dataset identifier PXD035726.

#### LC–MS/MS protein intensity quantification values

Peptides in the raw spectra were identified and quantified by MaxQuant label-free quantification software (version 1.6.17 for Windows)^35^ using UniProt proteome protein database for *Homo sapiens* (UP000005640 including isoforms, assembly 2021/02). Default tolerances for the Orbitrap instrument setting were used, i.e., 20 ppm for the first search and 4.5 ppm for the main peptide search at the MS level. Reverse sequences were selected for a target-decoy database strategy^36^, and a 1% false discovery rate was applied to both the peptide spectrum match and the protein group levels. Trypsin was chosen as a proteolytic enzyme, and two missed cleavages were allowed. The fixed modification was set to cysteine carbamidomethylation. Variable protein modifications were set to methionine oxidation, N-terminal protein acetylation, and methanol metabolism specific modifications—N-terminal protein Schiff base, formation of the imidazolidone group at the N-terminal valine (shift by one atom of C in both modifications), N-terminal protein formylation (shift by one atom of C and one atom of O). A maximum of five modifications per peptide was allowed. The minimum required peptide length was set to seven amino acids for successful identification, and only unique peptides were used for quantification. MaxQuant label-free quantification (LFQ) without the FastLFQ option was used for initial protein and peptide intensity normalization. Match between runs was switched on, and Dependent peptides search was also switched on using a 1% false discovery rate.

Protein intensity quantification values were computed from LFQ values of peptides, similar to^37,38^: For each unique peptide detected for a given protein, its LFQ value was divided by its peptide length, and a maximum of these values was used as the protein intensity quantification value. Shared peptides were not taken into account to avoid any additional unwanted variance. Proteins not identified by any MS/MS spectrum (“Only identified by site” column in the MaxQuant proteinGroups.txt file), proteins identified through the target-decoy database strategy (“Reverse” column in the MaxQuant proteinGroups.txt file), and pure contaminant proteins (“Potential contaminant” column in the MaxQuant proteinGroups.txt file; proteinGroups.txt containing only proteins with the prefix “CON__” in their identifiers) were removed from any subsequent analyses, as well as a few proteins identified by no unique peptide.

### Statistical analysis

#### Preliminary analysis

Statistical analysis was run in the R software (version 4.0.3 for Windows)^39^. To identify samples with a significantly lower number of identified proteins, the Kolmogorov–Smirnov test (α = 0.05) from the “stats” R package (version 4.0.3) was run to assess the normality for each of the sample groups M, S, and C, followed by the Grubb’s test (α = 0.05) from the “outliers” R package (version 0.14). For principal component analysis (PCA) of vectors of protein intensity quantification values, the multiMS-toolbox (version 2.6)^40,41^ for the R software was used.

#### Identification of proteins with significant changes in intensities

The Student’s t-test (α = 0.05) over protein intensity quantification values and the Benjamini-Hochberg^42^ correction (α = 0.05) for multiple comparisons for an aggregated number of tests over several comparisons were run using the default R package “stats”. The sum of tests was set to 1770 (3 × 590), while each protein was tested at most against values from three other sets (e.g., M vs. S, M vs. C, M vs. SC). Venn diagrams were plotted using the “ggvenn” package (version 0.1.9). Linear discriminant analysis (LDA) for the classification abilities of individual proteins was run on protein intensity quantification values using the R package “caret” (version 6.0.88). PCA-LDA and partial least squares discrimination analysis (PLS-DA) were run on vectors of protein intensity quantification values using the R packages “caret” and “pls” (version 2.7.3). The “oneSE” rule was applied to select the optimal number of PLS latent variables, and either leave-one-out cross-validation or repeated k-fold cross-validation schemes (k = 10, times = 3) were used. Pairwise-comparison of samples for the acute-poisoning phase and the long-term surviving phase.

For pairwise comparison of corresponding samples in groups M_pair_ and S_pair_, for each pair, the protein intensity quantification values for the sample from M_pair_ were subtracted from the protein intensity quantification values for the corresponding sample from S_pair_. The Student’s t-test was applied to test whether the difference in intensities significantly (α = 0.05) differs from zero. The Benjamini–Hochberg correction (α = 0.05) for multiple comparisons was applied subsequently.

#### Enrichment in gene ontology and pathway terms for selected groups

Protein information for all UniProt identifiers from the “Majority Protein IDs “ column of the MaxQuant proteinGroups.txt file was downloaded from MyGene.info Live API service (v3)^43^ (accessed 16th September 2021), and Gene Ontology (GO) terms^44^ were extracted using the R package “jsonlite” (version 1.7.2). To avoid testing the terms assigned in only a few cases, the subsequent overrepresentation analysis dealt only with such terms that occurred in the tested set in at least 10% of cases and in at least 4 occurrences. The Fisher’s exact test for count data for evaluating potential enrichment (α = 0.05) in GO terms was then run on all terms identified in the tested set of proteins in an occurrence ratio greater than the occurrence ratio for the full set of proteins (590 proteins) using the default R package “stats”, and the Benjamini–Hochberg correction (α = 0.05) for multiple comparisons was applied. Similarly, the Kyoto encyclopedia of genes and genomes (KEGG) pathways information^45^, Reactome information^46^, and WikiPathways information^47^ were also extracted and evaluated for any potential term enrichment in the tested sets of proteins. For KEGG pathways information, the terms generally occur at lower frequencies, and thus, the threshold was set to test terms that occurred in the tested set in at least 5% of cases and at least 4 occurrences.

#### Enrichment in KEGG hierarchical terms

The Kyoto encyclopedia of genes and genomes also provides a hierarchical classification for gene functions (BRITE, etc.). UniProt identifiers of genes from the “Majority Protein IDs “column of the MaxQuant proteinGroups.txt file were mapped to KEGG identifiers using the KEGG website API, and KEGG gene information was downloaded (KEGG Release 100.0+/11–29, Nov 21, accessed 29th November 2021). All KEGG hierarchical classifications were extracted using R for Windows. Due to a generally lower occurrence of terms, the subsequent overrepresentation analysis dealt only with terms occurring in the tested set in at least 5% of cases and at least 4 occurrences. The Fisher’s exact test for count data for evaluating potential enrichment (α = 0.05) was then run on all terms identified in the tested set of proteins in an occurrence ratio greater than twice that of the full set of proteins (590 proteins) using the default R package “stats”, and the Benjamini–Hochberg correction (α = 0.05) for multiple comparisons was applied.

### Ethics approval and consent to participate

The study was approved by the General University Hospital Ethics Committee (Decision No. 31/15) and was conducted in accordance with the principles of the Declaration of Helsinki. All patients who potentially qualified for the prospective study of long-term health sequelae of poisoning were informed about the study and its design. Only patients that provided their informed consent were recruited.

## Results

### Preliminary analysis

**Figure 1:**
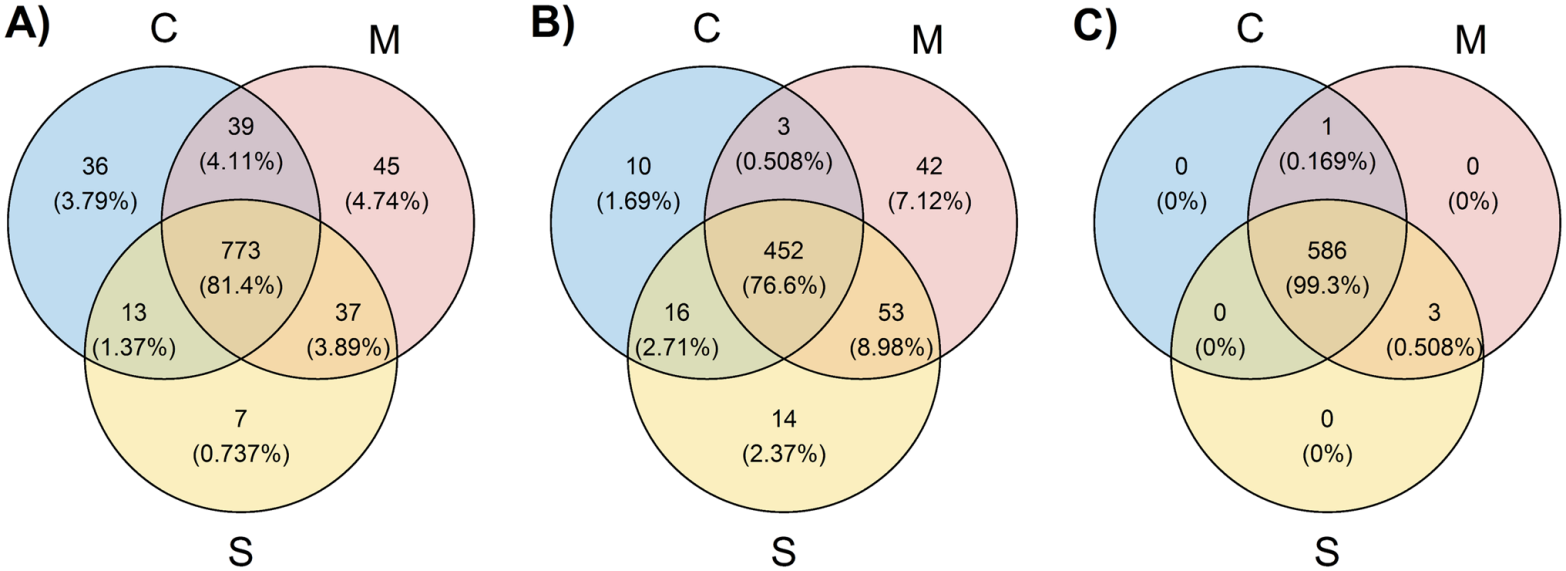
Proteins identified in selected groups. (**A**) All (950 in total) proteins identified; (**B**) proteins identified in at least 50% of samples in the given group (590); (**C**) proteins identified in at least 50% of the samples in at least one group of M, S, or C (590).

A total of 950 different proteins identified by at least one unique peptide (see Supplementary file 5) were detected in the blood serum samples (groups M, S, C, see also Table 1, Fig. 1). No significant difference was found in the number of proteins identified in each sample. Similarly, we can count how many samples each protein was identified in and then draw identified protein counts for a given sample count threshold. Visual inspection of such a histogram confirmed that for values from 50% of samples upwards, there is only a minimal decrease in the number of identified proteins for any of the three examined groups, with the sharp decrease only in the close neighborhood of a 100% samples ratio. The vast majority of identified proteins are common to all three groups (Fig. 1). If only proteins identified in at least 50% of samples in a given group are taken into account (590 in total, Fig. 1B), then behind the 452 proteins identified in all three groups, in addition to a large number of unique proteins identified in group M (42 proteins), there is also a large proportion of 53 proteins common to both—the group with acute poisoning M and the group of survivors S. The control group C then shows the smallest number of proteins identified in at least 50% of the group samples in all (481 proteins), with only small overlaps to group M alone or group S alone. For group C, many proteins were detected in only a few samples and thus are not included when a 50% threshold ratio is applied to identify proteins in the samples. These are, however, included when all the identified proteins in the group are accounted for regardless of how many samples were identified (see Fig. 1A) or when only proteins identified in at least 50% of any group are accounted for (see Fig. 1C), which was finally selected for subsequent quantitative and qualitative analyses (590 proteins in total, Fig. 1C). The vast majority of these proteins were detected in all groups. If they were not detected there in 50% or more samples in each of the groups (452 proteins), they were typically detected in at least 50% or more samples of either group M or both group M and group S. Due to selected high threshold ratio of proteins identified in samples the imputation of protein intensity values was omitted.

**Table 1:** Numbers of proteins identified in individual groups.

Proteins	Group	M	S	C
All	The total number of proteins identified in a given group	894	830	861
	The average number of proteins identified in the group samples*	559.46 ± 62.09	538.83 ± 23.17	505.00 ± 40.41
Proteins identified in at least 50% of the group samples	The total number of proteins identified in a given group	550	535	481
	The average number of proteins identified in the group samples*	491.88 ± 22.67	487.60 ± 10.84	437.46 ± 13.46
Proteins identified in at least 50% of the samples in any group of M, S, or C	The total number of proteins identified in a given group	590	589	587
	The average number of proteins identified in the group samples*	505.29 ± 26.41	503.30 ± 13.63	468.54 ± 22.31

M—patients with acute poisoning, S—long-term surviving patients, C—control group.

*The deviation represents one standard deviation.

Focusing on the protein intensities, Supplement Fig. 1 (Supplementary file 2) shows the PCA scores plot for the first two components for the vector of protein intensities identified in at least 50% of samples of any group of M, S, or C. The selected proteins include almost all the variance, and the PCA scores plot for the vector of intensities of all proteins (the original amount of 950 identified proteins) looks identical. The distribution of patients between groups M, S, and C is quite clearly visible. Thus, acute methanol poisoning and differences for the group of long-term surviving patients are the important factors determining the variance of the identified protein intensities in blood serum samples.

### Proteins with a significant change in intensity and their classification abilities

For 590 proteins with at least 50% representation in at least one group of M, S, or C, Table 2 shows the number of proteins with a significant change in intensity between the selected two groups (after application of multiple comparisons correction). Visual inspection of the histogram of *p* values of the t-tests confirmed that the distribution of samples between groups is the most important factor influencing the protein intensities (see Supplementary file 6 for a complete list of proteins with a significant change in intensity between groups).

**Table 2:** The number of proteins with significantly changed intensities between the selected two groups. The number of proteins with a significant change in protein intensity quantification values between the selected two groups, followed by five proteins with the most significant change according to the t-test *p* value from each comparison (a) M versus S, (b) M versus C, (c) M versus SC, (d) S versus C. The *p* value = *p* value of the Student’s t-test for the intensity quantification values of selected proteins; q value = t-test *p* value after the multiple comparison test correction by the Benjamini–Hochberg procedure. Fold change = binary logarithm of the ratio of mean protein intensity quantification value of tested groups. LDA accuracy for classification = linear discriminant analysis of the intensities of selected proteins using a leave-one-out cross-validation scheme to estimate the accuracy.

	M versus S	M versus C	M versus SC	S versus C
Total amount of proteins with a significant change in intensity	97	192	130	145

Protein	*p* value	*q* value	Fold change	LDA accuracy for classification (%)
**(a) M versus S**				
ITIH4_HUMAN Inter-alpha-trypsin inhibitor heavy chain H4^*^	2.38E−24	4.21E−21	− 2.19	98.57
B7ZKJ8_HUMAN Inter-alpha-trypsin inhibitor heavy chain H4^**^	9.42E−19	8.33E−16	− 2.26	95.71
KNG1_HUMAN Kininogen-1^***^	1.08E−17	6.40E−15	− 2.11	94.29
IPSP_HUMAN Plasma serine protease inhibitor	1.54E−16	6.83E−14	− 3.72	100.00
CO3_HUMAN Complement C3	3.29E−13	1.17E−10	3.70	98.57
**(b) M versus C**				
B7ZKJ8_HUMAN Inter-alpha-trypsin inhibitor heavy chain H4^**^	5.04E−16	8.92E−13	− 2.46	100.00
KNG1_HUMAN Kininogen-1^***^	4.39E−14	3.88E−11	−2.48	95.83
IPSP_HUMAN Plasma serine protease inhibitor	8.14E−14	4.80E−11	− 3.14	100.00
CO3_HUMAN Complement C3	2.31E−13	1.02E−10	4.02	97.92
VTNC_HUMAN Vitronectin	2.88E−13	1.02E−10	0.74	91.67
**(c) M versus SC**				
ITIH4_HUMAN Inter-alpha-trypsin inhibitor heavy chain H4 (Uniprot id = Q14624)^*^	1.80E−29	3.18E−26	− 2.31	98.94
B7ZKJ8_HUMAN Inter-alpha-trypsin inhibitor heavy chain H4 (other isoform, Uniprot id = B7ZKJ8)^**^	5.93E−29	5.25E−26	− 2.33	96.81
KNG1_HUMAN Kininogen-1^***^	9.66E−28	5.70E−25	− 2.25	96.81
IPSP_HUMAN Plasma serine protease inhibitor	5.52E−24	2.44E−21	− 3.55	98.94
CO3_HUMAN Complement C3	3.10E−13	1.10E−10	3.80	98.94
**(d) S versus C**				
MMRN2_HUMAN Multimerin-2	3.51E−17	6.22E−14	3.17	90.00
SAMP_HUMAN Serum amyloid P-component	5.70E−15	5.04E−12	1.20	87.14
Q5VY30_HUMAN Retinol-binding protein	1.30E−14	7.68E−12	0.98	90.00
PROC_HUMAN Vitamin K-dependent protein C	8.66E−14	3.62E−11	0.90	87.14
FA10_HUMAN Coagulation factor X	1.02E−13	3.62E−11	0.81	88.57

^*^MaxQuant search id 762.

^**^MaxQuant search id 1000,

^***^MaxQuant search id 181.

Due to the low values of the *p* values of the t-tests, some proteins themselves may serve as a discriminating factor alone (the marker) between the selected groups. To determine their classification ability, the accuracy of LDA classification of samples between groups was calculated only based on the detected protein intensity quantification values. When classified into only two classes, the achieved accuracy corresponds to the accuracy achieved with partial least squares—discriminant analysis (PLS-DA). All proteins found with 100% classification accuracy between the selected groups (classes) are included among the proteins listed in Table 2. For every two classes compared, all proteins with the highest classification accuracy between the classes are among the first five proteins from the differential analysis of the *p* values of the t-tests and are also included in Table 2. The only exception is for the classification between S and C groups. The classification accuracy between S and C reached 91.43% for the DIAC_HUMAN Di-N-acetylchitobiase (MaxQuant search id = 700) and the APOA_HUMAN Apolipoprotein A (MaxQuant search id = 356) proteins, which would be in the order of the *p* value of the t-test placed up to the 7th and 8th place.

Significant differences between the individual groups were highlighted by full discriminant analysis. Table 3 shows the accuracy achieved for the classification to groups using PCA-LDA and PLS-DA using the full set of proteins (590 proteins), not only the one. A similar high level of classification accuracy, as depicted in Table 3, is also maintained even if repeated k-fold cross-validation is used (k = 10, times = 3) instead of a leave-one-out cross-validation scheme. 100% classification accuracy is not always achieved; however, if the classification does not reach 100%, it still remains at 98.5% and above for PLS-DA for classification between any two or three groups tested. For PLS-DA classification and the repeated k-fold cross-validation scheme (but similarly for the leave-one-out scheme), for the final model trained on all patients of the classified groups, and for M vs. S classification, all the C samples were classified into group S. For S vs. C classification, all the M samples were classified to S. To classify M vs. C, 60.87% of the samples from S were classified to C and the rest to M.

**Table 3:** Accuracy of classification between groups using the full set of proteins (590 proteins).

Method	M versus S	M versus C	M versus SC	S versus C	M versus S versus C
PCA-LDA	100.00	97.92	98.94	97.14	97.87
	98.47	97.28	99.33	97.74	97.89
PLS-DA	100.00 (2)	100.00 (1)	100.00 (2)	98.57 (1)	98.94 (3)
	100.00 (2)	99.33 (1)	100.00 (2)	98.63 (1)	99.30 (3)

Accuracy of classification between groups using PCA-LDA or PLS-DA and leave-one-out cross-validation (top value) or repeated k-fold cross-validation (k = 10, times = 3) (bottom value). For PLS-DA, the number in parenthesis lists the number of latent variables required.

Supplement Fig. 2 (Supplementary file 3) shows the overlaps of sets of proteins with a significant change in protein intensity quantification values. To compare the M versus SC group, the detected proteins with a significant change can either be found among the proteins with a significant change in the comparison of M versus S or among the proteins with a significant change in the comparison of M versus C. If harder criteria should be met for proteins discriminating the acute-poisoning phase group M from the others, then some 64 proteins with a significant change in protein intensity quantification values still remain if we compare the group M to any of the S, C, and SC groups. For their list, see Supplementary file 6.

The overlap between the M versus S set, M versus C set, and S versus C set is also non-zero, containing 15 proteins that show a significant change between any two groups. Their list, together with the average protein intensity quantification values in groups, is given in Table 4. There is no protein alone in the selection that would be able to distinguish samples into all three classes with high accuracy (see Supplement Table 1 (Supplementary file 4)). However, for the successful classification of acute poisoning (i.e., high accuracy in M vs. S, M vs. C, M vs. SC), two proteins can be found in the given selection that are able to perform classification with high accuracy (see Supplement Table 1 (Supplementary file 4)) without the need to use any additional data. For IPSP_HUMAN, G3V2M1_HUMAN: Plasma serine protease inhibitor, the highest intensities were recorded in group S, followed by the intensities in group C, and the lowest intensities were measured in group M but still with non-zero values in all the group samples. For the other one, KNG1_HUMAN kininogen, MaxQuant divided all detected isoforms of kininogen into two different protein groups. In contrast, the high accuracy of classification to the group with acute poisoning versus to other groups is only seen in one of them (id = 181, UniProt id = P01042, see Supplement Table 1). This high accuracy is also followed by the low classification accuracy of distinguishing between the S and C groups. The between-group classification accuracy is more balanced for the other isoforms forming the other protein group (id = 182, Uniprot id P01042-2, P01042-3). If we combine both groups into one (all isoforms, id = 181 + 182), then the mean intensity levels between the groups will be even more differentiated. However, in terms of classification, for total kininogen, the high accuracy (95.83%) will remain only when distinguishing between group M and group C. The accuracy of the classification between group M and group S will decrease.

**Table 4:** Proteins with a significant change in intensity regardless of groups selected for comparison.

Protein	Increase or decrease in intensity (C → S → M)	Mean intensity in group C	Mean intensity in group S	Mean intensity in group M	Category of function
KNG1_HUMAN Kininogen-1 (UniProt id = P01042)*	↘	560E+07	435E+07	101E+07	Blood coagulation cascade
		± 156E+07	± 172E+07	± 574E+06	
KNG1_HUMAN Kininogen-1 (other isoforms, UniProt id = P01042-2, P02042-3)**	↘	558E+07	371E+07	231E+07	Blood coagulation cascade
		± 256E+07	± 133E+07	± 745E+06	
KNG1_HUMAN Kininogen-1 (all isoforms)	↘	112E+08	806E+07	332E+07	Blood coagulation cascade
		± 320E+07	± 281E+07	± 995E+06	
IPSP_HUMAN, G3V2M1_HUMAN: Plasma serine protease inhibitor	?	120E+07	180E+07	136E+06	Plasminogen cascade
		± 361E+06	± 895E+06	± 112E+06	
FGD6_HUMAN, F8VQX5_HUMAN, F8VY01_HUMAN: FYVE, RhoGEF, and PH domain-containing protein 6	↘	211E+07	645E+06	133E+06	JNK pathway
		± 169E+07	± 491E+06	± 249E+06	
AMBP_HUMAN, S4R3Y4_HUMAN: Protein AMBP; S4R471_HUMAN: Alpha-1-microglobulin	↗	920E+07	118E+08	165E+08	Heme degradation, immune system, signaling
		± 210E+07	± 254E+07	± 411E+07	
APOD_HUMAN, C9JF17_HUMAN: Apolipoprotein D	↗	395E+06	591E+06	105E+07	Lipid and vitamin A metabolism, neuroprotective effects
		± 143E+06	± 224E+06	± 476E+06	
CPN2_HUMAN Carboxypeptidase N subunit 2	↗	443E+07	617E+07	974E+07	Plasminogen cascade
		± 139E+07	± 265E+07	± 369E+07	
G3XAM2_HUMAN, CFAI_HUMAN, E7ETH0_HUMAN: Complement factor I; A0A2R8Y3M9_HUMAN: Uncharacterized protein	↗	371E+07	496E+07	614E+07	Immune system
		± 873E+06	± 988E+06	± 125E+07	
CD14_HUMAN, D6RFL4_HUMAN: Monocyte differentiation antigen CD14	↗	971E+06	144E+07	231E+07	Inflammation processes
		± 229E+06	± 504E+06	± 101E+07	
PRG4_HUMAN, J3KP74_HUMAN: Proteoglycan 4 (Lubricin)	↗	621E+06	104E+07	176E+07	Friction reduction
		± 232E+06	± 431E+06	± 857E+06	
VWF_HUMAN: von Willebrand factor	↗	281E+06	415E+06	134E+07	Blood coagulation cascade
		± 103E+06	± 165E+06	± 120E+07	
BTD_HUMAN: Biotinidase	↗	909E+06	122E+07	149E+07	Signaling
		± 216E+06	± 230E+06	± 340E+06	
A2AP_HUMAN, A0A0G2JPA8_HUMAN, C9JMH6_HUMAN , A0A0J9YY65_HUMAN: Alpha-2-antiplasmin	↗	775E+07	105E+08	134E+08	Plasminogen cascade
		± 342E+07	± 327E+07	± 354E+07	
Q5VY30_HUMAN, RET4_HUMAN: Retinol-binding protein	↗	145E+08	286E+08	382E+08	Vitamin A metabolism
		± 421E+07	± 781E+07	± 130E+08	
FINC_HUMAN Fibronectin	↗	105E+08	137E+08	179E+08	Plasminogen cascade
		± 244E+07	± 396E+07	± 550E+07	

Proteins with a significant change in protein intensity quantification values when comparing any two groups of C, S, and M, i.e., the set (M versus S) ∩ (M versus C) ∩ (S versus C) of 15 proteins. Protein intensity quantification values are given together with one standard deviation.

*MaxQuant search id 181,

**MaxQuant search id 182.

### Proteins with a significant change in intensity in a pairwise comparison of samples from the acute poisoning phase and the long-term surviving phase

For ten patients, serum samples were available both from admission to the hospital with acute poisoning (group M_pair_) and from the follow-up examination of survivors (group S_pair_). Comparing these samples allowed us to search for proteins with significant changes in intensities between the groups, filtering out individual differences of patients. The Student’s t-test revealed a total of 14 proteins that showed a significant change in intensity even after applying the correction to multiple testing (see Table 5). Most of these proteins (10 of 14) are present among the 64 proteins, with a significant change in protein intensity quantification values in the comparisons of group M to any other of the S, C, and SC groups. The remaining four are also detected as the proteins with a significant change in protein intensity quantification values in a comparison of group M to at least one group of S and SC. On the other hand, no significant change in protein intensity quantification values was detected for these four proteins in the comparison of M versus C.

**Table 5:** Proteins with a significant change in intensity in a pairwise comparison of samples.

Protein	*p* value	q value	Increase or decrease in intensity for M_pair_	t-test *p* value order in M versus S comparison	Member of proteins with a significant change in intensity
CO3_HUMAN: Complement C3	2.23E−07	1.31E−04	↑	5	M versus S, M versus C, M versus SC
TITIN_HUMAN, A0A0A0MTS7_HUMAN, A0A0A0MRA3_HUMAN, A0A1B0GXE3_HUMAN, H0Y4J7_HUMAN: Titin	5.98E−05	1.74E−02	↓	92	M versus S, M versus C, M versus SC
FETUA_HUMAN, C9JV77_HUMAN: Alpha-2-HS-glycoprotein	8.86E−05	1.74E−02	↓	16	M versus S, M versus C, M versus SC
ITIH4_HUMAN: Inter-alpha-trypsin inhibitor heavy chain H4*	1.78E−04	2.00E−02	↓	1	M versus S, M versus C, M versus SC
KNG1_HUMAN: Kininogen-1	2.10E−04	2.00E−02	↓	3	M versus S, M versus C, M versus SC
PLMN_HUMAN: Plasminogen	2.33E−04	2.00E−02	↓	9	M versus S, M versus C, M versus SC
FGFR1_HUMAN, B5A958_HUMAN, A0A3B3ISD1_HUMAN, E7EU09_HUMAN, C1KBH7_HUMAN, A0A6I8PRY1_HUMAN, E9PNM3_HUMAN, E9PN14_HUMAN: Fibroblast growth factor receptor 1	2.38E−04	2.00E−02	↓	18	M versus S, M versus SC
SRGN_HUMAN: Serglycin	3.73E−04	2.58E−02	↓	12	M versus S, M versus SC
IPSP_HUMAN, G3V2M1_HUMAN: Plasma serine protease inhibitor	3.94E−04	2.58E−02	↓	4	M versus S, M versus C, M versus SC
D6RAQ1_HUMAN, HPSE_HUMAN: Heparanase	6.04E−04	3.35E−02	↓	7	M versus S, M versus C, M versus SC
HYES_HUMAN, E5RFU2_HUMAN, H0YAW7_HUMAN: Bifunctional epoxide hydrolase 2	6.26E−04	3.35E−02	↓	41	M versus S
APOH_HUMAN, J3QRN2_HUMAN: Beta-2-glycoprotein 1	7.97E−04	3.91E−02	↓	25	M versus S, M versus SC
COL11_HUMAN: Collectin-11	9.98E−04	4.31E−02	↑	32	M versus S, M versus C, M versus SC
SEM4B_HUMAN, H0YMZ3_HUMAN, H0YN49_HUMAN, H0YMR1_HUMAN, H0YLN3_HUMAN, H0YMD6_HUMAN: Semaphorin-4B	1.02E−03	4.31E−02	↑	66	M versus S, M versus C, M versus SC

Proteins with a significant change in intensity in a group of 10 patients in a pairwise comparison of samples from the acute poisoning phase (M_pair_) and the long-term surviving phase (S_pair_).

*MaxQuant search id 762.

### Enrichment of GO, KEGG, Reactome, and WikiPathways terms in groups of proteins with significant changes

Previous comparisons between selected groups highlighted sets of proteins with significant changes in protein intensity quantification values. These sets were tested for potential overrepresentation of gene ontology terms (GO terms) separately in each of the commonly used categories—biological processes (BP), cellular component (CC), and molecular function (MF). First, GO terms were assigned for all tested proteins (590 proteins) for each inspected category. Then, for any previously identified set of proteins with a significant change in protein intensity quantification values, the Fisher’s exact test was performed for potential overrepresentation of terms assigned separately for each of the BP, CC, and MF GO categories (see Table 6 and Supplementary file 7). For the set of 64 proteins with a significant change in protein intensity quantification values in all comparisons related to the acute poisoning phase (in Table 6 marked as M vs. S ∩ M vs. C ∩ M vs. SC) or a set of 15 proteins showing a significant change in intensity in any comparison of groups M, S, and C (in Table 6 marked as M vs. S ∩ M vs. C ∩ S vs. C), then a significant predominance of terms related to the extracellular occurrence of proteins is clearly visible. The potential shift in peptidase activity is reported as the only potential overrepresentation term for biological processes and molecular functions. The detailed inspection of 15 proteins in M vs. S ∩ M vs. C ∩ S vs. C by a human researcher also showed an increased number of proteins from blood coagulation cascade and general peptidase activity, where no such enrichment in GO terms was detected. For the M vs. S ∩ M vs. C ∩ S vs. C set of proteins, the enrichment analysis was then also run on the aggregated occurrence of any GO terms containing the words “coagulation”, “peptidase”, and “endopeptidase”. For each of the mentioned words and in all three categories (BP, CC, MF), the significant enrichment was confirmed if there was at least one protein with a term containing the selected word, and thus, the enrichment analysis was also run using three freely accessible frequently cited pathway databases, Kyoto encyclopedia of genes and genomes (KEGG)^45^ including its hierarchical classification, Reactome^46^, and WikiPathways^47^. Table 6 and Supplement Table 2 (Supplementary file 4) show the terms from the mentioned databases with a significant enrichment in three previously tested sets of proteins—(M versus S) ∩ (M versus C) ∩ (M versus SC) (i.e. 64 proteins), (M versus S) ∩ (M versus C) ∩ (S versus C) (i.e. 15 proteins), and M_pair_ versus S_pair_ (pairwise, 14 proteins). For the full list of identified terms, including their *p* values and q values in all identified sets of proteins with significant changes in intensities, see Supplementary file 7. Using all three pathway databases, a significant overrepresentation is seen in complement and coagulation cascade proteins, fibrin formation pathways, and platelet and hemostasis activity.

**Table 6:** Significant overrepresentation of gene ontology terms and pathway terms in selected sets of proteins.

	(M versus S) ∩ (M versus C) ∩ (M versus SC)	(M versus S) ∩ (M versus C) ∩ (S versus C)	M_pair_ versus S_pair_
GO BP	*Involved_in negative regulation of endopeptidase activity* (GO:0010951, 13, 1.16E−02)	*Involved_in negative regulation of endopeptidase activity* (GO:0010951, 5, 4.25E−02)	*Involved_in negative regulation of endopeptidase activity* (GO:0010951, 5, 3.31E-02)
GO CC	*Located_in collagen−containing extracellular matrix* (GO: 0062023, 23, 3.08E−02), *is_active_in extracellular space* (GO: 0005615, 41, 3.08E−02), *located_in platelet alpha granule lumen* (GO: 0031093, 10, 3.08E−02), *extracellular region* (GO: 0005576, 44, 3.08E−02), *blood microparticle* (GO: 0072562, 16, 3.08E−02)	*Located_in extracellular region* (GO: 0005576, 14, 9.16E−03), *platelet alpha granule lumen* (GO: 0031093, 5, 9.16E−03), *is_active_in extracellular space* (GO: 0005615, 13, 9.16E−03), *located_in collagen−containing extracellular matrix* (GO: 0062023, 8, 9.16E−03)*, blood microparticle* (GO: 0072562, 6, 9.16E−03)	− *
GO MF	*Enables endopeptidase inhibitor activity* (GO:0004866, 10, 2.78E−04)	*Enables protease binding* (GO: 0002020, 4, 2.92E−02)	*Enables endopeptidase inhibitor activity* (GO:0,004,866, 4, 1.04E−02)
KEGG	*Neuroactive ligand-receptor interaction* (hsa04080, 6, 2.90E−03)**	*Complement and coagulation cascades* (hsa04610, 6, 2.49E−03)	*Complement and coagulation cascades* (hsa04610, 4, 4.94E−02)
Reactome	*Class A/1 (Rhodopsin-like receptors)* (R-HSA-373076, 7, 3.96E−04), *Peptide ligand-binding receptors* (R-HSA-375276, 7, 3.96E−04), *G alpha (i) signalling events* (R-HSA-418594, 7, 3.96E−04), *GPCR ligand binding* (R-HSA-500792, 7, 5.89E−04), *GPCR downstream signalling* (R-HSA-388396, 7, 8.61E−04), *Signaling by GPCR* (R-HSA-372790, 7, 1.23E−03), *Platelet degranulation* (R-HSA-114608, 15, 1.30E−02), *Response to elevated platelet cytosolic Ca*^*2*+^ (R-HSA-76005, 15, 1.30E−02), *Platelet activation, signaling and aggregation* (R-HSA-76002, 15, 2.55E−02)	*Intrinsic Pathway of Fibrin Clot Formation* (R-HSA-140837, 4, 8.30E−03), *Formation of Fibrin Clot (Clotting Cascade)* (R-HSA-140877, 4, 1.77E−02), *Platelet degranulation* (R-HSA-114608, 5, 4.87E−02), *Response to elevated platelet cytosolic Ca*^*2*+^ (R-HSA-76005, 5, 4.87E−02), *Hemostasis* (R-HSA-109582, 6, 4.87E−02), *Platelet activation, signaling and aggregation* (R-HSA-76002, 5, 4.87E−02)	*Platelet degranulation* (R-HSA-114608, 7, 1.85E−03), *Response to elevated platelet cytosolic Ca*^*2*+^ (R-HSA-76005, 7, 1.85E−03), *Platelet activation, signaling and aggregation* (R-HSA-76002, 7, 1.85E−03), *Hemostasis* (R-HSA-109582, 8, 1.85E−03)
WikiPathways	*Complement and Coagulation Cascades* (WP558, 10, 4.39E−02), *Complement system* (WP2806, 7, 3.10E−01)	*Complement and Coagulation Cascades* (WP558, 6, 5.38E−04)	*Complement and Coagulation Cascades* (WP558, 4, 1.96E−02)

Significant overrepresentation of gene ontology terms and pathway terms in selected sets of proteins with a significant change in intensity. For other tested sets of proteins, see Supplementary file 7. GO term or pathway term identifier, number of detected proteins, and q value for overrepresentation test are listed in parentheses. GO—Gene Ontology terms, BP—biological processes, CC—cellular component, MF—molecular function.

*Terms with the lowest q values are *located_in platelet alpha granule lumen* (GO: 0031093, 4) and *located_in extracellular region* (GO: 0005576, 12), yet their q values (both 7.40E−02) are above the 0.05 limit.

**Complement and coagulation cascades term here is the third one according to Fisher’s exact test *p* values, yet its q value here is 2.29E−01.

### Detection of potential N-terminal protein modifications originating from methanol

Methanol and its intermediate metabolites, like formaldehyde, are known to be responsible for protein structure modifications—especially N-terminal or side amino group modifications ^48^. To verify this potential modification, especially for hemoglobin, sequences for the α-, β-, and δ-subunits of hemoglobin commonly present in blood were downloaded from the UniProt database, and the MaxQuant software was set to search only for hemoglobin molecules and its potential N-terminal modifications. Even with this limitation, MaxQuant did not detect proteins with the N-terminal valine imidazolidone group and found only unmodified peptides or peptides with N-terminal acetylation (see details in Supplementary file 8).

### Identification of proteins with significant changes in (M versus S) ∩ (M versus C) ∩ (S versus C)

The 15 proteins (Table 4), which are significant among all three analyzed groups (M vs. S, M vs. C, S vs. C), can be divided into four groups according to their functions – coagulation and plasminogen cascades, immune defense, stress-activated protein kinase pathway, and metabolism of vitamin A. The detailed description of functions of identified proteins with relation to methanol intoxication can be found in Supplementary file 9. No relevant information was found only for proteoglycan 4 (lubricin, increased).

### Identification of proteins with significant changes in (M versus S) ∩ (M versus C) ∩ (M versus SC), and pairwise in M_pair_ versus S_pair_

Among the 64 proteins with significant changes in protein intensity quantification values in the comparisons of group M to any other group of S, C, or SC, Table 7 lists 15 proteins with the most significant changes (according to M vs. S q value) with an additional 5 proteins added to also cover all proteins with significant changes from the M_pair_ vs. S_pair_ comparison. For some of them (*Coagulation and plasminogen cascades*: kininogen-1, plasma serine protease inhibitor; *JNK pathway*: FYVE, RhoGEF, and PH domain-containing protein 6), detailed information on their functions was given above. The detailed description of functions of identified proteins with relation to methanol intoxication can be found in Supplementary file 10.

**Table 7:** The first 15 proteins with the most significant change in intensity (according to M versus S) from the intersection of M versus any other group comparisons.

Protein	Increase or decrease in intensity for M	Category of function	Significant change
ITIH4_HUMAN Inter-alpha-trypsin inhibitor heavy chain H4 (Uniprot id = Q14624)*	↓	Inflammation processes, Blood coagulation cascade, Plasminogen cascade	(M versus S) ∩ (M versus C) ∩ (M versus SC), Mpair versus Spair
B7ZKJ8_HUMAN Inter-alpha-trypsin inhibitor heavy chain H4 (other isoform, Uniprot id = B7ZKJ8)**	↓	Inflammation processes, Blood coagulation cascade, Plasminogen cascade	(M versus S) ∩ (M versus C) ∩ (M versus SC)
KNG1_HUMAN Kininogen-1 (UniProt id = P01042)***	↓	Blood coagulation cascade	(M versus S) ∩ (M versus C) ∩ (M versus SC), M_pair_ versus S_pair_
IPSP_HUMAN Plasma serine protease inhibitor	↓	Plasminogen cascade	(M versus S) ∩ (M versus C) ∩ (M versus SC), M_pair_ versus S_pair_
CO3_HUMAN Complement C3	↑	Inflammation processes	(M versus S) ∩ (M versus C) ∩ (M versus SC), M_pair_ versus S_pair_
CBPQ_HUMAN Carboxypeptidase Q	↓	Blood coagulation	(M versus S) ∩ (M versus C) ∩ (M versus SC)
COL11_HUMAN collectin-11	↑	Immune system	(M versus S) ∩ (M versus C) ∩ (M versus SC), M_pair_ versus S_pair_
ANGI_HUMAN Angiogenin	↓	Induces vascularization	(M versus S) ∩ (M versus C) ∩ (M versus SC)
D6RAQ1_HUMAN Heparanase isoform 3 of heparanase	↓	Regulates cell proliferation and angiogenesis	(M versus S) ∩ (M versus C) ∩ (M versus SC), M_pair_ versus S_pair_
PLMN_HUMAN Plasminogen	↓	Anticoagulant, Inflammation processes	(M versus S) ∩ (M versus C) ∩ (M versus SC), M_pair_ versus S_pair_
KNG1_HUMAN Kininogen-1 (other isoforms, UniProt id = P01042-2, P02042-3)****	↓	Blood pressure regulation	(M versus S) ∩ (M versus C) ∩ (M versus SC)
GELS_HUMAN Gelsolin	↓	Regulates the architecture and dynamics of cells	(M versus S) ∩ (M versus C) ∩ (M versus SC)
FETUA_HUMAN Alpha-2-HS-glycoprotein	↓	Endocytosis	(M versus S) ∩ (M versus C) ∩ (M versus SC), M_pair_ versus S_pair_
FGD6_HUMAN Isoform 2 of FYVE, RhoGEF and PH domain-containing protein 6	↓	JNK pathway	(M versus S) ∩ (M versus C) ∩ (M versus SC)
E7ES19_HUMAN Thrombospondin-4	↓	Blood coagulation	(M versus S) ∩ (M versus C) ∩ (M versus SC)
TITIN_HUMAN Titin	↓	Inflammation processes	(M versus S) ∩ (M versus C) ∩ (M versus SC), M_pair_ versus S_pair_
FGFR1_HUMAN Fibroblast growth factor receptor 1	↓	Cell proliferation and differentiation	M_pair_ versus S_pair_
SRGN_HUMAN Serglycin	↓	Cell apoptosis	M_pair_ versus S_pair_
HYES_HUMAN Bifunctional epoxide hydrolase 2	↓	Inflammation processes	M_pair_ versus S_pair_
APOH_HUMAN Beta-2-glycoprotein 1	↓	Complement and coagulation cascade	M_pair_ versus S_pair_
SEM4B_HUMAN Semaphorin-4B	↑	Synaptogenesis	(M versus S) ∩ (M versus C) ∩ (M versus SC), M_pair_ versus S_pair_

The first 15 proteins in (M versus S) ∩ (M versus C) ∩ (M versus SC) with the most significant change (according to M versus S q value) in protein intensity quantification values, with additional 5 remaining proteins with a significant change in protein intensity quantification values from comparison of M_pair_ versus S_pair_.

*MaxQuant search id 762,

**MaxQuant search id 1000,

***MaxQuant search id 181,

****MaxQuant search id 182.

#### Proteins with significant changes only in M versus S

Among the first 15 proteins with the most significant changes between M and S, almost all the proteins except three are also in (M versus S) ∩ (M versus C) ∩ (M versus SC) and were mentioned earlier. The remaining ones are protocadherin beta-12, serglycin, and interleukin-1 receptor accessory protein. All of these three are significantly decreased in M vs. S. and M vs. SC, but they are all increased in S versus C (sometimes even significantly). Close values for protein intensity quantification values in samples from M and C disqualify them to be significantly changed in (M versus S) ∩ (M versus C) ∩ (M versus SC). Functions of all these three proteins are also discussed in detail in Supplementary file 10.

### Identification of proteins with significant changes in concentrations between S and C groups

As in the previous chapter, the proteins responsible for the distribution between the S and C groups can be divided into similar subgroups—coagulation cascade, immune and inflammation response, and vitamin A metabolism and lipid transport. Table 8 shows 15 proteins with the most significant changes, containing also retinol-binding protein (increased) and apolipoprotein A (increased) mentioned earlier. The detailed description of functions of identified proteins with relation to methanol intoxication can be found in Supplementary file 11. No relevant information was found only for multimerin-2 (increased in M vs. C, S vs. C) and di-N-acetylchitobiase (increased).

**Table 8:** The first 15 proteins with the most significant changes in intensities in S versus C.

Protein	Increase or decrease in intensity for S	Category of function
MMRN2_HUMAN Multimerin-2	↑	Angiogenesis and tumor progression
SAMP_HUMAN Serum amyloid P-component	↑	Immune system, Bacterial infection response
Q5VY30_HUMAN Retinol-binding protein	↑	Vitamin A metabolism
PROC_HUMAN Vitamin K-dependent protein C	↑	Blood coagulation cascade
FA10_HUMAN Coagulation factor X	↑	Blood coagulation cascade
APOM_HUMAN Apolipoprotein M	↑	Enhances atheroprotective effects
DIAC_HUMAN Di-N-acetylchitobiase	↑	Inflammation processes
APOA_HUMAN Apolipoprotein(a)	↑	Inhibits tissue-type plasminogen activator 1
VTNC_HUMAN Vitronectin	↑	Plasminogen cascade
HEP2_HUMAN Heparin cofactor 2	↑	Blood coagulation
FA9_HUMAN Coagulation factor IX	↑	Blood coagulation cascade
K1522_HUMAN uncharacterized protein KIAA1522	↑	Unknown function
C9IZP8_HUMAN Complement C1s subcomponent	↑	Immune system
MASP1_HUMAN Mannan-binding lectin serine protease 1	↑	Immune system, Bacterial infection response
IC1_HUMAN Plasma protease C1 inhibitor	↑	Immune system, Blood coagulation cascade

The first 15 proteins with the most significant changes in protein intensity quantification values in a comparison of groups S and C.

## Discussion

In contrast to studies on ethanol-damaged liver^49,50^ and brain tissue^51^, in our study, which focused on blood plasma analysis, changes in different proteins were detected. On the other side, the detected sets of proteins overlap with ethanol-related studies targeted for blood plasma, such as for the detection of alcoholic acute pancreatitis^52^. Similarly, for methanol intoxication studies, the set of proteins with differential expression in dissimilar tissues like rat retina^23^ is also different and unrelated to our results on blood samples. Finding biomarkers for methanol/ethanol intoxication in blood plasma is more complicated due to the blood circulation, faster reactions in blood plasma, and mainly due to the previous patient treatment.

The results obtained from the group of proteins acting in blood coagulation and plasminogen cascades (kininogen 1, von Willebrand factor, alpha-2-antiplasmin, plasma serine protease inhibitor, carboxypeptidase N, fibronectin, plasminogen, coagulation factors, etc.) show a general increased tendency for coagulation for the methanol poisoning group vs. any other group. Blood coagulation, however, is also affected by the use of heparin administration during hemodialysis. Nevertheless, the increased tendency for coagulation still prevails even in comparison between groups of survivors and the control group. Heparin requires a plasma cofactor for its anticoagulant activity—antithrombin III^53,54^. The heparin-antithrombin III complex inactivates several coagulation enzymes, including thrombin factor (IIa) and coagulation factors X_a_, IX_a_, XI_a_, and XII_a_^55^. Of these, thrombin and factor X_a_ are the most responsive to inhibition. By inactivating thrombin, heparin not only prevents fibrin formation but also inhibits thrombin-induced activation of coagulation factor V and factor VIII^56–58^. Unfractionated heparin also binds von Willebrand factor (vWF) and inhibits the vWF–platelet glycoprotein Ib (GP Ib) interaction^59^. Even though the von Willebrand factor is affected by heparin treatment, its significant increase could also be affected by the methanol intoxication alone (significant differences in concentrations among any two compared groups, including between survivors and the control group). vWf is known to be increased in several other health-affecting conditions like obesity^60^ or in 30 days follow-up of patients with unstable angina with end point event registered (death, myocardial infarction, revascularization) than in patients free of such events^61^. Thus, it can be an important marker even for methanol intoxication. Due to the heparin administration and several blood sampling procedures on admission to the hospital, we cannot clearly confirm whether for blood coagulation, the methanol intoxication is similar to excessive alcohol consumption. More recent studies also suggest that intakes of up to one drink per day do not influence the plasminogen activator inhibitor-1 (PAI-1) concentration, but intakes higher than this increase the PAI-1 concentration^62,63^ and activity^64^. Plasma tissue plasminogen activator (tPA) antigen was reported also to be increased in heavy drinkers, which is consistent with a decreased fibrinolytic activity^65^. The acute withdrawal of ethanol is known to have rebound effects, including the activation of platelets by normal agonists^66^; therefore, while moderate alcohol consumption can be protective against cardiovascular disease partly through decreased fibrinogen levels, short-term binge drinking may have a hypofibrinolytic effect through increased PAI-1 levels and rebound platelet hyperaggregability^67^.

Previous animal studies have shown that methanol intoxication can affect the neuroimmune system’s function and induce specific and non-specific systemic immune responses, probably mainly by increasing oxidative stress and secondary changes in corticosterone levels^68–70^. The neuroinflammatory and immune response could also result from brain lesions^25^ and potential blood**–**brain barrier damage, subsequently amplified by the complement system^16–18^. Our results confirm increased markers of immune response (e.g., increased complement factor I, complement factors C3 and C5, monocyte differentiation antigen CD14, and collectins). From results on C3- and C5-deficient mice, complement factors C3 and C5 probably play an important protective role for neurons against death caused by excitotoxins^71,72^. Further analysis suggested their influence on the signaling cascade resulting in caspase-3 downregulation, thereby reducing apoptotic cell death^73^. Complement seems to be also involved in brain injury-induced neurogenesis. After transient ischemia, C3-deficient mice showed a significant reduction in neurogenesis compared to wildtype mice^74^. Ischemic neonatal mice treated with C3a exhibited improved memory over C3a-receptor-deficient neonatal mice, and, in C3a overexpressing mice, hypoxia–ischemia-induced tissue loss was reduced by 50% when compared to wildtype mice^75^.

The increased concentrations of lipid metabolism proteins—apolipoprotein A I, apolipoprotein A II, adiponectin, etc., reported here (both increased in the methanol poisoning group in comparison to the control group) are similar to other studies^76^ for moderate ethanol consumption. Our results also show a significant increase in the retinol-binding protein (RBP) in the blood serum of the methanol poisoning group in comparison to the group of survivors or the control group. Similarly, a significant increase for RBP is also visible in the comparison of the group of survivors to the control group. The relationship between vitamin A metabolism and ethanol consumption is known from studies on mutant mice^77,78^. Decreased plasma retinol levels and plasma retinol binding protein concentrations have been reported in alcoholics by several groups^79–81^. Leo and Lieber^82^ then showed that a significant decrease in the hepatic retinoid content (correlated with disease severity) is only observed in those patients with more advanced stages of disease (hepatitis and cirrhosis). Furthermore, separate quantification of hepatic retinol and retinyl ester concentrations found that both retinoid species were decreased in the livers of alcoholics^83–85^. The increased concentrations of RBP in our study are in contrast to previous ethanol-consumption based studies. It is not known whether these higher levels of retinol-binding protein concentrations during methanol intoxication are only a side-effect of broken retinol transport and metabolism in retinal epithelium cells, other retina damage, or some kind of protective effect (see^86–90^).

Even though no specific marker for discrimination of methanol poisoning and ethanol intoxication was found, the most interesting candidates could be proteins of the interleukin-1 beta system, here, the interleukin-1 receptor accessory protein. In the methanol poisoning group, this protein was significantly reduced, resulting probably in effects similar to knockout of its associate, interleukin-1 receptor, i.e., reduced sensitivity to the sedative effects of ethanol and increased severity of acute ethanol withdrawal^91^, which could serve as a body response or signal of acute poisoning in hopes of changing the organism behavior and immediately stopping ethanol/methanol consumption.

No found methanol-related N-terminal protein modifications in identified proteins is not in contradiction with literature^92^, where especially for this task, a targeted approach with a heavy labeled standard and Selective Reaction Monitoring type of analysis and detection was used. The sensitivity of such methods is usually several orders of magnitude higher in comparison with our untargeted approach. Moreover, the mentioned study aimed at peptides originating from hemoglobin and red blood cells, which would only be unwanted contamination in our samples.

## Conclusion

Based on the obtained results, the effect of acute methanol intoxication on the proteomic composition of blood serum cannot be unambiguously determined, partially because of the influence of heparin during hemodialysis. However, proteins closely related to intoxication have been identified, mainly those involved in blood coagulation, immune response or inflammation (e.g., complement factor I, complement factors C3 and C5), and metabolism of vitamin A (increase in retinol-binding protein). Apolipoprotein A I and apolipoprotein A II (and other lipid metabolism proteins like adiponectin) also show a significant increase.

For blood-coagulation, the most affected proteins with significant changes in the methanol poisoning group were von Willebrand factor, carboxypeptidase N, alpha-2-antiplasmin (all increased), inter-alpha-trypsin inhibitor heavy chain H4, kininogen-1, plasma serine protease inhibitor, and plasminogen (all decreased). However, changes in the concentration of some of them could be interfered with by heparin administration.

If any markers for discrimination of acute methanol poisoning vs. methanol-free ethanol intoxication should be searched for, then above changes in concentration of retinol-binding protein, the most interesting candidates could be proteins of the interleukin-1 beta system. Here, the interleukin-1 receptor accessory protein was found with significantly decreased levels in patients with acute methanol poisoning in comparison to other patient samples.

## Supplementary Information

Supplementary Information 1.

Supplementary Information 2.

Supplementary Information 3.

Supplementary Information 4.

Supplementary Information 5.

Supplementary Information 6.

Supplementary Information 7.

Supplementary Information 8.

Supplementary Information 9.

Supplementary Information 10.

Supplementary Information 11.

## Data Availability

The acquired mass spectrometry proteomics data were deposited to the ProteomeXchange Consortium via the PRIDE^34^ partner repository with the dataset identifier PXD035726 including lists of found peptides and proteins. The list of proteins used for analyses and the complete list of proteins with a significant change in intensity between groups, both in Excel format, are also available in the supplement.

## Abbreviations

ADH: Alcohol dehydrogenase
AMBP: Alpha-1*-*microglobulin, bikunin
APC: Activated form of protein C
ApoD: Apolipoprotein D
ApoM: Apolipoprotein M
ATP: Adenosine triphosphate
BP: Biological processes
C3: Complement C3
CC: Cellular component
CD14: Monocyte differentiation antigen CD14
Cdc42: Cell division control protein 42
CPN: Carboxypeptidase N
FN: Fibronectin
FX: Coagulation factor X
GO: Gene ontology
HCD: Higher energy collisional dissociation
HDL: High-density lipoprotein
HMWK: High-molecular-weight-kininogen
HS: Heparanase
IL-1β: Interleukin-1 beta system
ITIH4: Inter-alpha-trypsin inhibitor heavy chain H4
JNK: C-Jun N-terminal kinase
KEGG: Kyoto encyclopedia of genes and genomes
LC–MS/MS: Liquid chromatography-tandem mass spectrometry
LDA: Linear discriminant analysis
LFQ: Label-free quantification
LMWK: Low-molecular-weight-kininogen
MAP3K: Mitogen-activated protein kinase kinase kinase
MAP4K: Mitogen-activated protein kinase kinase kinase kinase
MAPK: Mitogen-activated protein kinase
mCD14: Membrane-bound CD14
MF: Molecular function
MS: Mass spectrometry
MS/MS: Tandem mass spectrometry
nLC-MS/MS: Nano-liquid chromatography-tandem mass spectrometry
PAI-1: Plasminogen activator inhibitor-1
PCA: Principal component analysis
PCA-LDA: Linear discriminant analysis over principal components
PCI: Protein C inhibitor, plasma serine protease inhibitor/
PLS-DA: Partial least squares discrimination analysis
RA: Retinoic acid
RBP: Retinol-binding protein
RoDH: Retinol dehydrogenase
sCD14: Soluble CD14
SD: Standard deviation
SDC: Sodium deoxycholate
SERPINs: Serine protease inhibitors
TCEP: Tris(2-carboxyethyl)phosphine hydrochloride
TEAB: Triethylammonium bicarbonate
TFA: Trifluoroacetic acid
tPA: Tissue-type plasminogen activators
uPA: Urokinase-type plasminogen activators
VWF: Von Willebrand factor
β2GPI: Beta-2-glycoprotein
