# Supplementary material for: Acute and chronic blood serum proteome changes in patients with methanol poisoning
Source: Sci Rep. 2022 Dec 9;12:21379. doi: 10.1038/s41598-022-25492-9 (PMC9734099; doi:10.1038/s41598-022-25492-9)
Supplement: Supplementary file 1 — Supplementary Information 1. [file 41598_2022_25492_MOESM1_ESM.pdf]

The content of supplementary information section

Supplementary information 1: This file.

Supplementary information 2: Supplement Figure 1. PCA scores plot for the vector of protein intensities identified in at least 50% of samples in at least one group of M, S, or C (590 proteins). M – patients with acute poisoning; M_{pair} – patients with acute poisoning and a related sample included in S; S – long-term surviving patients; S_{pair} – long-term surviving patients and a related sample included in M; C – control group.

Supplementary information 3: Supplement Figure 2. The overlaps of sets of proteins with a significant change in protein intensity quantification values.

Supplementary information 4:

> **Supplement Table 1.** LDA accuracy for classification between selected groups for proteins with significant change in protein intensity quantification values when comparing any two groups of C, S, and M, i.e., proteins from the set M vs. S ∩ M vs. C ∩ S vs. C (15 proteins). LDA is applied on the intensity quantification values of selected proteins using a leave-one-out cross-validation scheme for estimation of the classification accuracy.
>
> **Supplement Table 2**. Significant overrepresentation of KEGG hierarchical classification terms in selected sets of proteins with a significant change of intensity. For more details and enrichment analysis in other tested sets, see **Supplementary information 6**.

Supplementary information 5: MS Excel file with 950 identified proteins, their normalized intensities, identified peptide counts, and FASTA headers. Proteins not identified by any MS/MS spectrum ("Only identified by site" column in the MaxQuant proteinGroups.txt file), proteins identified through the target-decoy database strategy ("Reverse" column in the MaxQuant proteinGroups.txt file), and pure contaminant proteins ("Potential contaminant" column in the MaxQuant proteinGroups.txt file; proteinGroups.txt containing only proteins with prefix "CON__" in their identifiers), and proteins identified by no unique peptide were removed. MaxQuant original search results files are also included.

Supplementary information 6: Complete list of proteins with a significant change in intensity between selected groups (M vs. S, M vs. C, M vs. SC, S vs. C, M_{pair} vs. S_{pair}) and some intersections of selected results (M vs. S ∩ M vs. C ∩ M vs. SC, M vs. S ∩ M vs. C ∩ S vs. C).

Supplementary information 7: Enrichment analysis of GO, KEGG, Reactome, and WikiPathways terms assigned to proteins with significant changes between groups. All terms above relative (at least in 5% or 10% of all cases) and absolute (at least in 4 occurrences in all) threshold are listed, and the significantly enriched terms (after Benjamini-Hochberg correction ($\alpha = 0.05$, *q-value* column)) are highlighted.

Supplementary information 8: Detailed search for potential N-terminal protein modifications originating from methanol and the results with their analysis.

Supplementary information 9: The detailed description of functions of identified proteins with significant changes in M vs. S ∩ M vs. C ∩ S vs. C.

Supplementary information 10: The detailed description of functions of identified proteins with significant changes in M vs. S ∩ M vs. C ∩ M vs. SC, and pairwise in M_{pair} vs. S_{pair}.

Supplementary information 11: The detailed description of functions of identified proteins with significant changes in in concentrations between S and C groups.
