# Supplementary material for: Acute and chronic blood serum proteome changes in patients with methanol poisoning
Source: Sci Rep. 2022 Dec 9;12:21379. doi: 10.1038/s41598-022-25492-9 (PMC9734099; doi:10.1038/s41598-022-25492-9)

Supplement Figure 1. PCA scores plot for the vector of protein intensities for samples from the M, S, and C groups. PCA scores plot for the vector of protein intensities identified in at least 50% of samples in at least one group of M, S, or C (590 proteins). M– patients with acute poisoning; M_{pair} – patients with acute poisoning and a related sample included in S; S – long-term surviving patients; S_{pair} – long-term surviving patients and a related sample included in M; C – control group.

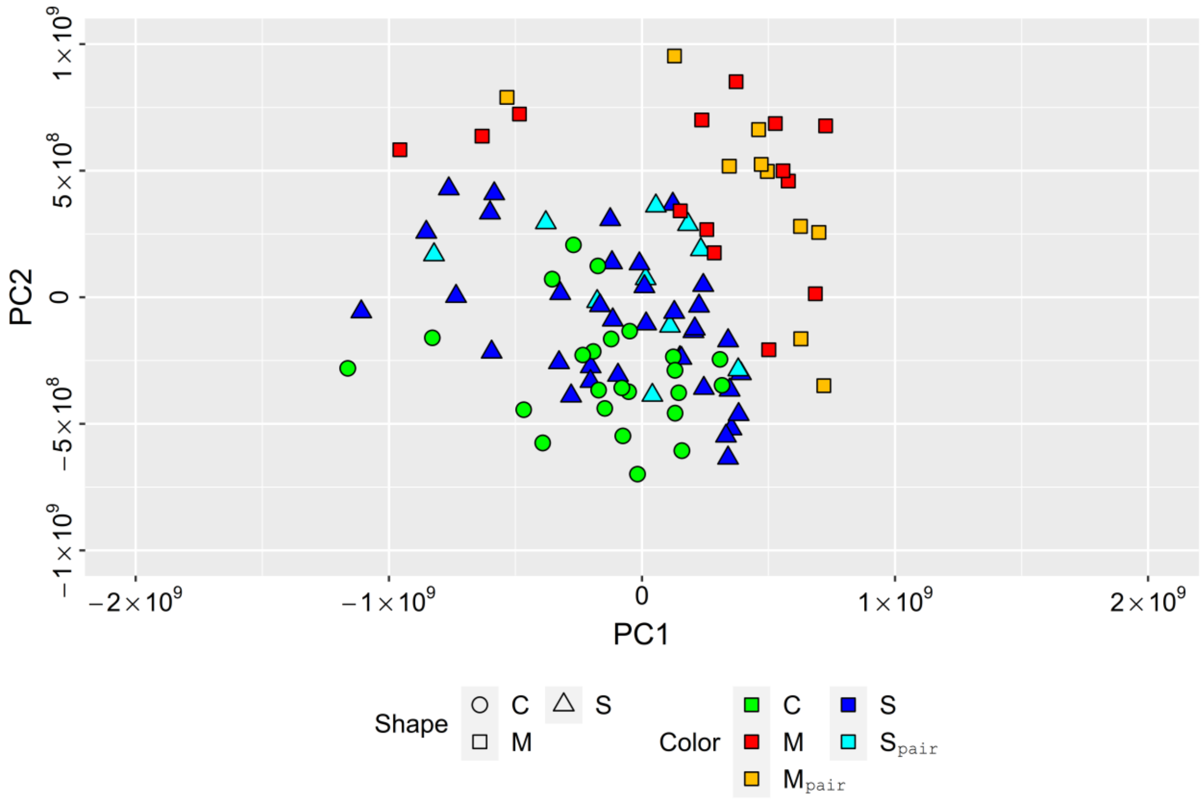

Supplement: Supplementary file 2 — Supplementary Information 2. [file 41598_2022_25492_MOESM2_ESM.pdf]
