# Supplementary material for: Acute and chronic blood serum proteome changes in patients with methanol poisoning
Source: Sci Rep. 2022 Dec 9;12:21379. doi: 10.1038/s41598-022-25492-9 (PMC9734099; doi:10.1038/s41598-022-25492-9)

Supplement Figure 2. The overlaps of sets of proteins with a significant change in protein intensity quantification values.

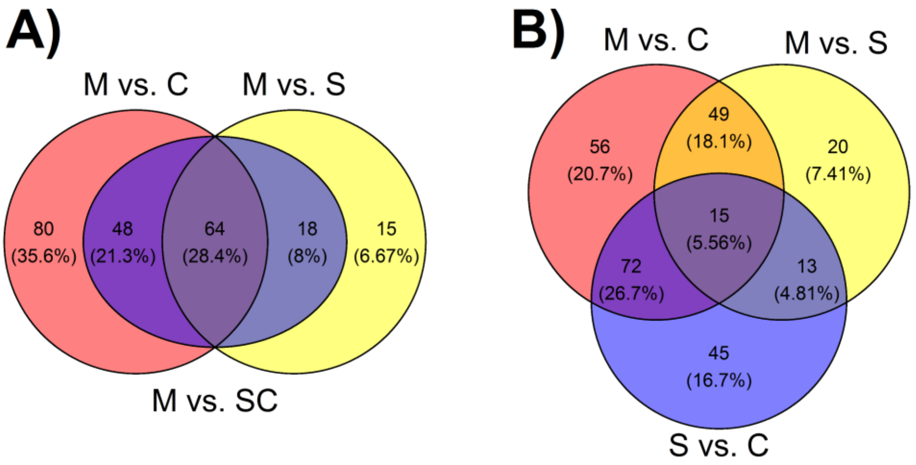

Supplement: Supplementary file 3 — Supplementary Information 3. [file 41598_2022_25492_MOESM3_ESM.pdf]
