# Supplementary material for: Acute and chronic blood serum proteome changes in patients with methanol poisoning
Source: Sci Rep. 2022 Dec 9;12:21379. doi: 10.1038/s41598-022-25492-9 (PMC9734099; doi:10.1038/s41598-022-25492-9)
Supplement: Supplementary file 4 — Supplementary Information 4. [file 41598_2022_25492_MOESM4_ESM.pdf]

## Supplement Table 1

**Supplement Table 1. LDA accuracy for classification between selected groups (using single proteins).** LDA accuracy for classification between selected groups for proteins with a significant change in protein intensity quantification values when comparing any two groups of C, S, and M, i.e., proteins from the set M vs. S ∩ M vs. C ∩ S vs. C (15 proteins). LDA is applied to intensity quantification values of selected single proteins using a leave-one-out cross-validation scheme to estimate classification accuracy.

| **Protein** | **LDA accuracy for classification** | | | | |
|---|---|---|---|---|---|
| | **M vs. S** | **M vs. C** | **M vs. SC** | **S vs. C** | **M vs. S vs. C** |
| KNG1_HUMAN Kininogen-1 (UniProt id=P01042)* | 94.29 | 95.83 | 96.81 | 62.86 | 72.34 |
| KNG1_HUMAN Kininogen-1 (other isoforms, UniProt id=P01042-2, P02042-3)** | 72.86 | 85.42 | 75.53 | 71.43 | 53.19 |
| KNG1_HUMAN Kininogen-1 (all isoforms) | 80.00 | 95.83 | 85.11 | 67.14 | 62.77 |
| FINC_HUMAN Fibronectin | 70.00 | 85.42 | 77.66 | 61.43 | 55.32 |
| Q5VY30_HUMAN, RET4_HUMAN: Retinol-binding protein | 70.00 | 87.50 | 76.60 | 90.00 | 72.34 |
| AMBP_HUMAN, S4R3Y4_HUMAN: Protein AMBP; S4R471_HUMAN: Alpha-1-microglobulin | 77.14 | 83.33 | 84.04 | 75.71 | 61.70 |
| VWF_HUMAN: von Willebrand factor | 75.71 | 77.08 | 81.91 | 65.71 | 57.45 |
| APOD_HUMAN, C9JF17_HUMAN: Apolipoprotein D | 77.14 | 85.42 | 82.98 | 75.71 | 55.32 |
| IPSP_HUMAN, G3V2M1_HUMAN: Plasma serine protease inhibitor | 100.00 | 100.00 | 98.94 | 65.71 | 77.66 |
| G3XAM2_HUMAN, CFAI_HUMAN, E7ETH0_HUMAN: Complement factor I; A0A2R8Y3M9_HUMAN: Uncharacterized protein | 75.71 | 87.50 | 80.85 | 74.29 | 64.89 |
| CD14_HUMAN, D6RFL4_HUMAN: Monocyte differentiation antigen CD14 | 72.86 | 83.33 | 79.79 | 70.00 | 54.26 |
| A2AP_HUMAN, A0A0G2JPA8_HUMAN, C9JMH6_HUMAN , A0A0J9YY65_HUMAN: Alpha-2-antiplasmin | 65.71 | 81.25 | 74.47 | 74.29 | 55.32 |
| CPN2_HUMAN Carboxypeptidase N subunit 2 | 74.29 | 79.17 | 81.91 | 64.29 | 54.26 |
| BTD_HUMAN: Biotinidase | 71.43 | 87.50 | 78.72 | 75.71 | 61.70 |
| FGD6_HUMAN, F8VQX5_HUMAN, F8VY01_HUMAN: FYVE, RhoGEF and PH domain-containing protein 6 | 81.43 | 79.17 | 74.47 | 82.86 | 62.77 |
| PRG4_HUMAN, J3KP74_HUMAN: Proteoglycan 4 | 71.43 | 83.33 | 78.72 | 75.71 | 55.32 |

*MaxQuant search id 181, **MaxQuant search id 182

## Supplement Table 2

**Supplement Table 2. Significant overrepresentation of KEGG hierarchical classification terms.** Significant overrepresentation of KEGG hierarchical classification terms in selected sets of proteins with a significant change in intensity. For more details and enrichment analysis in other tested sets, see **Supplement File S3**.

| KEGG hierarchical term (including path) | Number of detected proteins | q-value |
|---|---|---|
| **M vs. S ∩ M vs. C ∩ M vs. SC** | | |
| KEGG Orthology (KO) [BR:hsa00001]:09130 Environmental Information Processing:09133 Signaling molecules and interaction:04080 Neuroactive ligand-receptor interaction | 5 | 3.58E-02 |
| Membrane trafficking [BR:hsa04131]:Endocytosis:Phagocytosis:Opsonins | 5 | 4.60E-02 |
| Peptidases and inhibitors [BR:hsa01002]:Peptidase inhibitors:Family I25: cystatin family | 4 | 4.60E-02 |
| Peptidases and inhibitors [BR:hsa01002]:Peptidase inhibitors | 9 | 4.60E-02 |
| KEGG Orthology (KO) [BR:hsa00001]:09130 Environmental Information Processing:09133 Signaling molecules and interaction | 13 | 4.60E-02 |
| **M vs. S ∩ M vs. C ∩ S vs. C** | | |
| Peptidases and inhibitors [BR:hsa01002]:Peptidase inhibitors | 5 | 1.50E-02 |
| KEGG Orthology (KO) [BR:hsa00001]:09150 Organismal Systems:09151 Immune system:04610 Complement and coagulation cascades | 6 | 1.67E-02 |
| KEGG Orthology (KO) [BR:hsa00001]:09180 Brite Hierarchies:09181 Protein families: metabolism:01002 Peptidases and inhibitors [BR:hsa01002] | 7 | 3.66E-02 |
| Peptidases and inhibitors [BR:hsa01002] | 7 | 3.66E-02 |
| KEGG Orthology (KO) [BR:hsa00001]:09160 Human Diseases:09174 Infectious disease: parasitic | 4 | 4.60E-02 |
| **$M_{pair}$ vs. $S_{pair}$** | | |
| KEGG Orthology (KO) [BR:hsa00001]:09180 Brite Hierarchies:09181 Protein families: metabolism | 8 | 3.06E-02 |
| Peptidases and inhibitors [BR:hsa01002]:Peptidase inhibitors | 4 | 3.06E-02 |
| KEGG Orthology (KO) [BR:hsa00001]:09180 Brite Hierarchies:09183 Protein families: signaling and cellular processes | 12 | 4.25E-02 |
| KEGG Orthology (KO) [BR:hsa00001]:09160 Human Diseases:09161 Cancer: overview | 5 | 4.25E-02 |
| KEGG Orthology (KO) [BR:hsa00001]:09180 Brite Hierarchies:09181 Protein families: metabolism:01002 Peptidases and inhibitors [BR:hsa01002] | 6 | 4.25E-02 |
| Peptidases and inhibitors [BR:hsa01002] | 6 | 4.25E-02 |
