# Supplementary material for: Acute and chronic blood serum proteome changes in patients with methanol poisoning
Source: Sci Rep. 2022 Dec 9;12:21379. doi: 10.1038/s41598-022-25492-9 (PMC9734099; doi:10.1038/s41598-022-25492-9)
Supplement: Supplementary file 7 — Supplementary Information 7. [file 41598_2022_25492_MOESM7_ESM.pdf]

Enrichment analysis

GO terms

Only terms that occurred in the tested set in at least 10% of cases and at least 4 occurrences. The Fisher's exact test for count data for evaluating potential enrichment (α = 0.05, *p-value* column) in GO terms was then run on all terms identified in the tested set of proteins in an occurrence ratio greater than that of the full set of proteins (590 proteins). The Benjamini-Hochberg correction (α = 0.05, *q-value* column) for multiple comparisons was applied (over tests in BP, CC, and MF categories). The q-values below or equal to 0.05 are highlighted in red, and values between 0.05 and 0.15 are highlighted in orange.

M vs. S

GO term	*count*	*p-value*	*q-value*	*qualifier*	*term.name*	*GO category*
GO:0010951	13	3.04E-02	6.98E-01	involved_in	negative regulation of endopeptidase activity	BP
GO:0006954	10	6.46E-02	7.43E-01	involved_in	inflammatory response	BP
GO:0007165	11	1.98E-01	1.00E+00	involved_in	signal transduction	BP

GO term	*count*	*p-value*	*q-value*	*qualifier*	*term.name*	*GO category*
GO:0031093	14	3.14E-03	7.23E-02	located_in	platelet alpha granule lumen	CC
GO:0005576	64	6.53E-03	7.50E-02	located_in	extracellular region	CC
GO:0005615	58	1.44E-02	8.94E-02	is_active_in	extracellular space	CC
GO:0062023	29	1.55E-02	8.94E-02	located_in	collagen-containing extracellular matrix	CC
GO:0031012	10	4.55E-02	2.09E-01	is_active_in	extracellular matrix	CC
GO:0072562	19	5.73E-02	2.20E-01	located_in	blood microparticle	CC
GO:0005788	18	1.22E-01	4.01E-01	located_in	endoplasmic reticulum lumen	CC
GO:0005783	10	2.96E-01	7.77E-01	located_in	endoplasmic reticulum	CC
GO:0009986	15	3.04E-01	7.77E-01	located_in	cell surface	CC
GO:0005886	33	4.40E-01	8.49E-01	located_in	plasma membrane	CC
GO:0070062	63	4.40E-01	8.49E-01	located_in	extracellular exosome	CC
GO:0005925	11	4.43E-01	8.49E-01	located_in	focal adhesion	CC

GO term	*count*	*p-value*	*q-value*	*qualifier*	*term.name*	*GO category*
GO:0004866	10	6.77E-04	1.56E-02	enables	endopeptidase inhibitor activity	MF
GO:0008201	12	2.40E-02	1.90E-01	enables	heparin binding	MF
GO:0005178	10	2.48E-02	1.90E-01	enables	integrin binding	MF
GO:0005102	11	1.29E-01	7.43E-01	enables	signaling receptor binding	MF
GO:0005515	74	1.85E-01	8.35E-01	enables	protein binding	MF
GO:0042803	11	2.18E-01	8.35E-01	enables	protein homodimerization activity	MF
GO:0005509	17	3.87E-01	1.00E+00	enables	calcium ion binding	MF

GO:0042802	16	5.44E-01	1.00E+00	enables	identical protein binding	MF

M vs. C

GO term	count	p-value	q-value	qualifier	term.name	GO category
GO:0010951	25	1.84E-03	1.84E-02	involved_in	negative regulation of endopeptidase activity	BP
GO:0006508	25	1.09E-01	5.44E-01	involved_in	proteolysis	BP

GO term	count	p-value	q-value	qualifier	term.name	GO category
GO:0072562	40	7.31E-04	6.64E-03	located_in	blood microparticle	CC
GO:0062023	55	1.33E-03	6.64E-03	located_in	collagen-containing extracellular matrix	CC
GO:0005576	119	4.68E-03	1.56E-02	located_in	extracellular region	CC
GO:0005615	109	6.48E-03	1.62E-02	is_active_in	extracellular space	CC
GO:0005783	20	1.54E-01	3.08E-01	located_in	endoplasmic reticulum	CC
GO:0005788	30	2.91E-01	4.84E-01	located_in	endoplasmic reticulum lumen	CC
GO:0070062	123	4.91E-01	6.46E-01	located_in	extracellular exosome	CC
GO:0009986	26	5.17E-01	6.46E-01	located_in	cell surface	CC

GO term	count	p-value	q-value	qualifier	term.name	GO category

M vs. SC

GO term	count	p-value	q-value	qualifier	term.name	GO category
GO:0010951	19	2.35E-03	3.76E-02	involved_in	negative regulation of endopeptidase activity	BP
GO:0007155	16	1.81E-01	1.00E+00	involved_in	cell adhesion	BP
GO:0006508	14	5.11E-01	1.00E+00	involved_in	proteolysis	BP

GO term	count	p-value	q-value	qualifier	term.name	GO category
GO:0005576	83	7.21E-03	7.99E-02	located_in	extracellular region	CC
GO:0005615	75	1.75E-02	7.99E-02	is_active_in	extracellular space	CC
GO:0072562	26	1.88E-02	7.99E-02	located_in	blood microparticle	CC
GO:0031093	15	2.01E-02	7.99E-02	located_in	platelet alpha granule lumen	CC
GO:0062023	36	2.50E-02	7.99E-02	located_in	collagen-containing extracellular matrix	CC
GO:0009986	23	7.14E-02	1.90E-01	located_in	cell surface	CC
GO:0005783	14	1.86E-01	4.26E-01	located_in	endoplasmic reticulum	CC
GO:0005788	21	2.81E-01	5.63E-01	located_in	endoplasmic reticulum lumen	CC
GO:0016021	13	4.68E-01	8.32E-01	located_in	integral component of membrane	CC

GO term	count	p-value	q-value	qualifier	term.name	GO category
GO:0005178	13	1.16E-02	1.86E-01	enables	integrin binding	MF
GO:0008201	13	9.11E-02	7.29E-01	enables	heparin binding	MF
GO:0042802	22	4.80E-01	1.00E+00	enables	identical protein binding	MF
GO:0005509	21	5.40E-01	1.00E+00	enables	calcium ion binding	MF

S vs. C

GO term	count	p-value	q-value	qualifier	term.name	GO category
GO:0007596	15	1.76E-03	2.63E-02	involved_in	blood coagulation	BP
GO:0006508	22	2.83E-02	2.13E-01	involved_in	proteolysis	BP
GO:0010951	17	4.39E-02	2.19E-01	involved_in	negative regulation of endopeptidase activity	BP
GO:0045087	15	1.95E-01	7.32E-01	involved_in	innate immune response	BP

GO term	count	p-value	q-value	qualifier	term.name	GO category
GO:0005576	89	2.60E-02	2.89E-01	located_in	extracellular region	CC
GO:0072562	27	4.22E-02	2.89E-01	located_in	blood microparticle	CC
GO:0005615	80	5.77E-02	2.89E-01	is_active_in	extracellular space	CC
GO:0062023	37	8.01E-02	3.01E-01	located_in	collagen-containing extracellular matrix	CC
GO:0005783	16	1.36E-01	4.08E-01	located_in	endoplasmic reticulum	CC
GO:0016020	30	3.12E-01	7.79E-01	located_in	membrane	CC
GO:0016021	15	3.97E-01	8.50E-01	located_in	integral component of membrane	CC

GO term	count	p-value	q-value	qualifier	term.name	GO category
GO:0004252	17	5.54E-03	8.32E-02	enables	serine-type endopeptidase activity	MF
GO:0005102	15	1.49E-01	1.00E+00	enables	signaling receptor binding	MF
GO:0042803	15	2.76E-01	1.00E+00	enables	protein homodimerization activity	MF
GO:0005509	25	3.77E-01	1.00E+00	enables	calcium ion binding	MF

M vs. S ∩ M vs. C ∩ M vs. SC

GO term	count	p-value	q-value	qualifier	term.name	GO category
GO:0010951	13	6.45E-04	1.16E-02	involved_in	negative regulation of endopeptidase activity	BP
GO:0006954	8	3.67E-02	3.30E-01	involved_in	inflammatory response	BP
GO:0007165	8	1.74E-01	1.00E+00	involved_in	signal transduction	BP

GO term	count	p-value	q-value	qualifier	term.name	GO category

GO:0062023	23	2.54E-03	3.08E-02	located_in	collagen-containing extracellular matrix	CC
GO:0005615	41	8.12E-03	3.08E-02	is_active_in	extracellular space	CC
GO:0031093	10	8.16E-03	3.08E-02	located_in	platelet alpha granule lumen	CC
GO:0005576	44	8.45E-03	3.08E-02	located_in	extracellular region	CC
GO:0072562	16	8.55E-03	3.08E-02	located_in	blood microparticle	CC
GO:0031012	7	7.24E-02	2.17E-01	is_active_in	extracellular matrix	CC
GO:0005788	12	1.81E-01	4.65E-01	located_in	endoplasmic reticulum lumen	CC
GO:0005783	7	2.91E-01	6.24E-01	located_in	endoplasmic reticulum	CC
GO:0070062	43	3.21E-01	6.24E-01	located_in	extracellular exosome	CC
GO:0009986	10	3.47E-01	6.24E-01	located_in	cell surface	CC

GO term	*count*	*p-value*	*q-value*	*qualifier*	*term.name*	*GO category*
GO:0004866	10	1.54E-05	2.78E-04	enables	endopeptidase inhibitor activity	MF
GO:0008201	9	2.44E-02	2.20E-01	enables	heparin binding	MF
GO:0005102	9	5.54E-02	3.32E-01	enables	signaling receptor binding	MF
GO:0005509	12	3.24E-01	1.00E+00	enables	calcium ion binding	MF
GO:0005515	47	4.60E-01	1.00E+00	enables	protein binding	MF

M vs. S ∩ M vs. C ∩ S vs. C

GO term	*count*	*p-value*	*q-value*	*qualifier*	*term.name*	*GO category*
GO:0010951	5	4.25E-03	4.25E-02	involved_in	negative regulation of endopeptidase activity	BP

GO term	*count*	*p-value*	*q-value*	*qualifier*	*term.name*	*GO category*
GO:0005576	14	1.21E-03	9.16E-03	located_in	extracellular region	CC
GO:0031093	5	2.27E-03	9.16E-03	located_in	platelet alpha granule lumen	CC
GO:0005615	13	2.75E-03	9.16E-03	is_active_in	extracellular space	CC
GO:0062023	8	5.24E-03	1.31E-02	located_in	collagen-containing extracellular matrix	CC
GO:0072562	6	1.06E-02	2.13E-02	located_in	blood microparticle	CC
GO:0070062	13	4.85E-02	8.08E-02	located_in	extracellular exosome	CC

GO term	*count*	*p-value*	*q-value*	*qualifier*	*term.name*	*GO category*
GO:0002020	4	2.92E-03	2.92E-02	enables	protease binding	MF
GO:0008201	4	1.57E-02	7.84E-02	enables	heparin binding	MF
GO:0005515	11	5.86E-01	1.00E+00	enables	protein binding	MF

M_{pair} vs. S_{pair}

GO term	*count*	*p-value*	*q-value*	*qualifier*	*term.name*	*GO category*
GO:0010951	5	3.01E-03	3.31E-02	involved_in	negative regulation of endopeptidase activity	BP

GO term	*count*	*p-value*	*q-value*	*qualifier*	*term.name*	*GO category*
GO:0031093	4	1.21E-02	7.40E-02	located_in	platelet alpha granule lumen	CC
GO:0005576	12	1.34E-02	7.40E-02	located_in	extracellular region	CC
GO:0072562	5	3.31E-02	1.21E-01	located_in	blood microparticle	CC
GO:0062023	6	5.23E-02	1.44E-01	located_in	collagen-containing extracellular matrix	CC
GO:0005615	9	1.91E-01	4.20E-01	is_active_in	extracellular space	CC
GO:0005886	5	5.11E-01	9.36E-01	located_in	plasma membrane	CC
GO:0070062	9	6.03E-01	9.47E-01	located_in	extracellular exosome	CC

GO term	*count*	*p-value*	*q-value*	*qualifier*	*term.name*	*GO category*
GO:0004866	4	9.47E-04	1.04E-02	enables	endopeptidase inhibitor activity	MF
GO:0008201	4	1.21E-02	6.66E-02	enables	heparin binding	MF
GO:0005515	11	4.18E-01	1.00E+00	enables	protein binding	MF

KEGG pathways terms

Only terms that occurred in the tested set in at least 5% of cases and in at least 4 occurrences in all. The Fisher's exact test for count data for evaluating potential enrichment ($\alpha = 0.05$, *p-value* column) in KEGG pathways terms was then run on all terms identified in the tested set of proteins in an occurrence ratio greater than that of the full set of proteins (590 proteins). The Benjamini-Hochberg correction ($\alpha = 0.05$, *q-value* column) for multiple comparisons was applied. q-values below or equal to 0.05 are highlighted in red, and values between 0.05 and 0.15 are highlighted in orange.

M vs. S

KEGG term	*count*	*p-value*	*q-value*	*term description (Homo sapiens domain)*
hsa04080	7	2.09E-04	3.35E-03	Neuroactive ligand-receptor interaction
hsa04918	5	7.93E-03	6.34E-02	Thyroid hormone synthesis
hsa04810	7	3.22E-02	1.72E-01	Regulation of actin cytoskeleton
hsa05014	5	4.67E-02	1.87E-01	Amyotrophic lateral sclerosis
hsa05203	5	6.33E-02	1.91E-01	Viral carcinogenesis
hsa04151	8	7.15E-02	1.91E-01	PI3K-Akt signaling pathway
hsa04610	14	1.18E-01	2.51E-01	Complement and coagulation cascades
hsa05205	6	1.36E-01	2.51E-01	Proteoglycans in cancer
hsa05020	6	1.60E-01	2.51E-01	Prion disease
hsa04512	6	1.87E-01	2.51E-01	ECM-receptor interaction
hsa05133	5	1.88E-01	2.51E-01	Pertussis
hsa05165	5	1.88E-01	2.51E-01	Human papillomavirus infection

hsa04510	6	2.44E-01	2.89E-01	Focal adhesion
hsa04145	5	2.53E-01	2.89E-01	Phagosome
hsa05200	6	3.05E-01	3.26E-01	Pathways in cancer
hsa05150	6	3.69E-01	3.69E-01	Staphylococcus aureus infection

M vs. C

KEGG term	*count*	*p-value*	*q-value*	*term description (Homo sapiens domain)*
hsa04610	29	9.67E-03	4.84E-02	Complement and coagulation cascades
hsa04145	10	1.05E-01	2.08E-01	Phagosome
hsa05171	15	1.25E-01	2.08E-01	Coronavirus disease - COVID-19
hsa05020	10	1.79E-01	2.24E-01	Prion disease
hsa05150	11	3.77E-01	3.77E-01	Staphylococcus aureus infection

M vs. SC

KEGG term	*count*	*p-value*	*q-value*	*term description (Homo sapiens domain)*
hsa04080	7	1.44E-03	1.73E-02	Neuroactive ligand-receptor interaction
hsa04610	20	3.27E-02	1.96E-01	Complement and coagulation cascades
hsa04151	10	6.49E-02	2.30E-01	PI3K-Akt signaling pathway
hsa05165	7	1.00E-01	2.30E-01	Human papillomavirus infection
hsa05020	8	1.09E-01	2.30E-01	Prion disease
hsa04810	7	1.27E-01	2.30E-01	Regulation of actin cytoskeleton
hsa04512	8	1.34E-01	2.30E-01	ECM-receptor interaction
hsa05205	7	1.90E-01	2.57E-01	Proteoglycans in cancer
hsa04510	8	1.92E-01	2.57E-01	Focal adhesion
hsa05171	10	2.22E-01	2.66E-01	Coronavirus disease - COVID-19
hsa05150	8	3.32E-01	3.63E-01	Staphylococcus aureus infection
hsa05200	7	4.23E-01	4.23E-01	Pathways in cancer

S vs. C

KEGG term	*count*	*p-value*	*q-value*	*term description (Homo sapiens domain)*
hsa04610	25	2,76E-03	5,52E-03	Complement and coagulation cascades - Homo sapiens (human)
hsa05171	10	3,48E-01	3,48E-01	Coronavirus disease - COVID-19 - Homo sapiens (human)

M vs. S ∩ M vs. C ∩ M vs. SC

KEGG term	*count*	*p-value*	*q-value*	*term description (Homo sapiens domain)*
hsa04080	6	1.93E-04	2.90E-03	Neuroactive ligand-receptor interaction
hsa05142	4	1.60E-02	1.20E-01	Chagas disease
hsa04610	11	5.77E-02	2.29E-01	Complement and coagulation cascades
hsa04145	5	6.61E-02	2.29E-01	Phagosome
hsa05020	5	9.22E-02	2.29E-01	Prion disease
hsa05150	6	9.41E-02	2.29E-01	Staphylococcus aureus infection

hsa04512	5	1.07E-01	2.29E-01	ECM-receptor interaction
hsa05133	4	1.40E-01	2.34E-01	Pertussis
hsa05165	4	1.40E-01	2.34E-01	Human papillomavirus infection
hsa04810	4	1.62E-01	2.41E-01	Regulation of actin cytoskeleton
hsa04151	5	1.77E-01	2.41E-01	PI3K-Akt signaling pathway
hsa05205	4	2.08E-01	2.60E-01	Proteoglycans in cancer
hsa04510	4	3.08E-01	3.50E-01	Focal adhesion
hsa05171	5	3.27E-01	3.50E-01	Coronavirus disease - COVID-19
hsa05200	4	3.61E-01	3.61E-01	Pathways in cancer

M vs. S ∩ M vs. C ∩ S vs. C

KEGG term	*count*	*p-value*	*q-value*	*term description (Homo sapiens domain)*
hsa04610	6	2.49E-03	2.49E-03	Complement and coagulation cascades

M_{pair} vs. S_{pair}

KEGG term	*count*	*p-value*	*q-value*	*term description (Homo sapiens domain)*
hsa04610	4	4.94E-02	4.94E-02	Complement and coagulation cascades

Reactome pathways terms

Only terms that occurred in the tested set in at least 10% of cases and in at least 4 occurrences in all. The Fisher's exact test for count data for evaluating potential enrichment (α = 0.05, *p-value* column) in Reactome pathways terms was then run on all terms identified in the tested set of proteins in an occurrence ratio greater than that of the full set of proteins (590 proteins). The Benjamini-Hochberg correction (α = 0.05, *q-value* column) for multiple comparisons was applied. q-values below or equal to 0.05 are highlighted in red, and values between 0.05 and 0.15 are highlighted in orange.

M vs. S

Reactome term	*count*	*p-value*	*q-value*	*term description*
R-HSA-114608	21	2.73E-03	1.26E-02	Platelet degranulation
R-HSA-76005	21	2.73E-03	1.26E-02	Response to elevated platelet cytosolic Ca2+
R-HSA-76002	22	3.45E-03	1.26E-02	Platelet activation, signaling and aggregation
R-HSA-162582	26	2.82E-02	7.74E-02	Signal Transduction
R-HSA-109582	24	3.82E-02	8.41E-02	Hemostasis
R-HSA-1430728	20	2.39E-01	4.24E-01	Metabolism
R-HSA-597592	20	2.70E-01	4.24E-01	Post-translational protein modification
R-HSA-1474244	11	3.49E-01	4.61E-01	Extracellular matrix organization
R-HSA-392499	25	3.77E-01	4.61E-01	Metabolism of proteins
R-HSA-381426	10	5.16E-01	5.23E-01	Regulation of Insulin-like Growth Factor (IGF) transport and uptake by Insulin-like Growth Factor Binding Proteins (IGFBPs)
R-HSA-1643685	17	5.23E-01	5.23E-01	Disease

M vs. C

Reactome term	*count*	*p-value*	*q-value*	*term description*
R-HSA-166658	21	1.13E-02	5.64E-02	Complement cascade
R-HSA-114608	29	8.81E-02	1.47E-01	Platelet degranulation
R-HSA-76005	29	8.81E-02	1.47E-01	Response to elevated platelet cytosolic Ca2+
R-HSA-76002	30	1.43E-01	1.79E-01	Platelet activation, signaling and aggregation
R-HSA-109582	37	2.94E-01	2.94E-01	Hemostasis

M vs. SC

Reactome term	*count*	*p-value*	*q-value*	*term description*
R-HSA-114608	22	4.68E-02	1.09E-01	Platelet degranulation
R-HSA-76005	22	4.68E-02	1.09E-01	Response to elevated platelet cytosolic Ca2+
R-HSA-166658	14	5.48E-02	1.09E-01	Complement cascade
R-HSA-76002	23	6.25E-02	1.09E-01	Platelet activation, signaling and aggregation
R-HSA-1474244	15	2.77E-01	3.88E-01	Extracellular matrix organization
R-HSA-109582	25	3.57E-01	4.16E-01	Hemostasis
R-HSA-1266738	15	4.96E-01	4.96E-01	Developmental Biology

S vs. C

Reactome term	*count*	*p-value*	*q-value*	*term description*
R-HSA-166658	16	3.08E-02	2.15E-01	Complement cascade
R-HSA-1430728	31	1.09E-01	2.83E-01	Metabolism
R-HSA-109582	31	1.21E-01	2.83E-01	Hemostasis
R-HSA-114608	20	2.94E-01	4.06E-01	Platelet degranulation
R-HSA-76005	20	2.94E-01	4.06E-01	Response to elevated platelet cytosolic Ca2+
R-HSA-76002	21	3.48E-01	4.06E-01	Platelet activation, signaling and aggregation
R-HSA-1643685	26	4.51E-01	4.51E-01	Disease

M vs. S ∩ M vs. C ∩ M vs. SC

Reactome term	*count*	*p-value*	*q-value*	*term description*
R-HSA-373076	7	6.60E-05	3.96E-04	Class A/1 (Rhodopsin-like receptors)
R-HSA-375276	7	6.60E-05	3.96E-04	Peptide ligand-binding receptors
R-HSA-418594	7	6.60E-05	3.96E-04	G alpha (i) signalling events
R-HSA-500792	7	1.31E-04	5.89E-04	GPCR ligand binding
R-HSA-388396	7	2.39E-04	8.61E-04	GPCR downstream signalling
R-HSA-372790	7	4.10E-04	1.23E-03	Signaling by GPCR
R-HSA-114608	15	5.77E-03	1.30E-02	Platelet degranulation

R-HSA-76005	15	5.77E-03	1.30E-02	Response to elevated platelet cytosolic Ca2+
R-HSA-76002	15	1.28E-02	2.55E-02	Platelet activation, signaling and aggregation
R-HSA-166658	8	7.18E-02	1.29E-01	Complement cascade
R-HSA-109582	16	8.09E-02	1.32E-01	Hemostasis
R-HSA-162582	16	1.38E-01	2.08E-01	Signal Transduction
R-HSA-597592	14	2.40E-01	3.27E-01	Post-translational protein modification
R-HSA-392499	18	2.54E-01	3.27E-01	Metabolism of proteins
R-HSA-381426	8	3.01E-01	3.28E-01	Regulation of Insulin-like Growth Factor (IGF) transport and uptake by Insulin-like Growth Factor Binding Proteins (IGFBPs)
R-HSA-1280218	7	3.10E-01	3.28E-01	Adaptive Immune System
R-HSA-8957275	7	3.10E-01	3.28E-01	Post-translational protein phosphorylation
R-HSA-1474244	7	4.45E-01	4.45E-01	Extracellular matrix organization

M vs. S ∩ M vs. C ∩ S vs. C

Reactome term	*count*	*p-value*	*q-value*	*term description*
R-HSA-140837	4	1.04E-03	8.30E-03	Intrinsic Pathway of Fibrin Clot Formation
R-HSA-140877	4	4.43E-03	1.77E-02	Formation of Fibrin Clot (Clotting Cascade)
R-HSA-114608	5	2.65E-02	4.87E-02	Platelet degranulation
R-HSA-76005	5	2.65E-02	4.87E-02	Response to elevated platelet cytosolic Ca2+
R-HSA-109582	6	3.48E-02	4.87E-02	Hemostasis
R-HSA-76002	5	3.65E-02	4.87E-02	Platelet activation, signaling and aggregation
R-HSA-1643685	5	9.91E-02	1.13E-01	Disease
R-HSA-162582	5	1.40E-01	1.40E-01	Signal Transduction

M_{pair} vs. S_{pair}

Reactome term	*count*	*p-value*	*q-value*	*term description*
R-HSA-114608	7	5.18E-04	1.85E-03	Platelet degranulation
R-HSA-76005	7	5.18E-04	1.85E-03	Response to elevated platelet cytosolic Ca2+
R-HSA-76002	7	8.66E-04	1.85E-03	Platelet activation, signaling and aggregation
R-HSA-109582	8	9.23E-04	1.85E-03	Hemostasis
R-HSA-381426	4	4.21E-02	6.74E-02	Regulation of Insulin-like Growth Factor (IGF) transport and uptake by Insulin-like Growth Factor Binding Proteins (IGFBPs)
R-HSA-162582	4	2.72E-01	3.62E-01	Signal Transduction
R-HSA-392499	4	4.46E-01	5.10E-01	Metabolism of proteins

R-HSA-168249	4	5.65E-01	5.65E-01	Innate Immune System

WikiPathways terms

Only terms that occurred in the tested set in at least 10% of cases and in at least 4 occurrences in all. The Fisher's exact test for count data for evaluating potential enrichment (α = 0.05, *p-value* column) in WikiPathways terms was then run on all terms identified in the tested set of proteins in an occurrence ratio greater than that of the full set of proteins (590 proteins). The Benjamini-Hochberg correction (α = 0.05, *q-value* column) for multiple comparisons was applied. q-values below or equal to 0.05 are highlighted in red, and values between 0.05 and 0.15 are highlighted in orange.

M vs. S

WikiPathways term	*count*	*p-value*	*q-value*	*term description*
WP558	12	6.60E-02	1.98E-01	Complement and Coagulation Cascades
WP45	10	2.72E-01	3.19E-01	G1 to S cell cycle control
WP2806	10	3.19E-01	3.19E-01	Complement system

M vs. C

WikiPathways term	*count*	*p-value*	*q-value*	*term description*
WP558	23	1.11E-02	2.22E-02	Complement and Coagulation Cascades
WP2806	21	1.12E-01	1.12E-01	Complement system

M vs. SC

WikiPathways term	*count*	*p-value*	*q-value*	*term description*
WP45	16	4.93E-02	1.39E-01	G1 to S cell cycle control
WP2806	16	6.96E-02	1.39E-01	Complement system
WP558	14	1.26E-01	1.66E-01	Complement and Coagulation Cascades
WP23	13	1.66E-01	1.66E-01	B Cell Receptor Signaling Pathway

S vs. C

WikiPathways term	*count*	*p-value*	*q-value*	*term description*
WP558	22	4.46E-04	4.46E-04	Complement and Coagulation Cascades

M vs. S ∩ M vs. C ∩ M vs. SC

WikiPathways term	*count*	*p-value*	*q-value*	*term description*
WP558	10	2.20E-02	4.39E-02	Complement and Coagulation Cascades
WP2806	7	3.10E-01	3.10E-01	Complement system

M vs. S ∩ M vs. C ∩ S vs. C

WikiPathways term	*count*	*p-value*	*q-value*	*term description*
WP558	6	5.38E-04	5.38E-04	Complement and Coagulation Cascades

M_{pair} vs. S_{pair}

WikiPathways term	*count*	*p-value*	*q-value*	*term description*
WP558	4	1.96E-02	1.96E-02	Complement and Coagulation Cascades

KEGG hierarchical classification terms

Only terms that occurred in the tested set in at least 5% of cases and in at least 4 occurrences in all. The Fisher's exact test for count data for evaluating potential enrichment (α = 0.05, *p-value* column) in KEGG pathways terms was then run on all terms identified in the tested set of proteins in an occurrence ratio greater than twice the ratio for the full set of proteins (590 proteins). The Benjamini-Hochberg correction (α = 0.05, *q-value* column) for multiple comparisons was applied. q-values below or equal to 0.05 are highlighted in red, and values between 0.05 and 0.15 are highlighted in orange.

M vs. S

KEGG hierarchical term	*count*	*p-value*	*q-value*
KEGG Orthology (KO) [BR:hsa00001]:09130 Environmental Information Processing:09133 Signaling molecules and interaction:04080 Neuroactive ligand-receptor interaction	6	9.46E-04	1.80E-02
KEGG Orthology (KO) [BR:hsa00001]:09150 Organismal Systems:09152 Endocrine system:04918 Thyroid hormone synthesis	5	7.93E-03	7.53E-02
Membrane trafficking [BR:hsa04131]:Endocytosis:Phagocytosis:Opsonins	5	2.20E-02	1.02E-01
KEGG Orthology (KO) [BR:hsa00001]:09130 Environmental Information Processing:09133 Signaling molecules and interaction	16	2.34E-02	1.02E-01
KEGG Orthology (KO) [BR:hsa00001]:09140 Cellular Processes:09142 Cell motility	7	3.22E-02	1.02E-01
KEGG Orthology (KO) [BR:hsa00001]:09140 Cellular Processes:09142 Cell motility:04810 Regulation of actin cytoskeleton	7	3.22E-02	1.02E-01
KEGG Orthology (KO) [BR:hsa00001]:09160 Human Diseases:09164 Neurodegenerative disease:05014 Amyotrophic lateral sclerosis	5	4.67E-02	1.27E-01
KEGG Orthology (KO) [BR:hsa00001]:09160 Human Diseases:09161 Cancer: overview:05203 Viral carcinogenesis	5	6.33E-02	1.50E-01
KEGG Orthology (KO) [BR:hsa00001]:09130 Environmental Information Processing:09132 Signal transduction:04151 PI3K-Akt signaling pathway	8	7.15E-02	1.51E-01
Peptidases and inhibitors [BR:hsa01002]:Peptidase inhibitors	9	8.76E-02	1.61E-01
KEGG Orthology (KO) [BR:hsa00001]:09160 Human Diseases:09167 Endocrine and metabolic disease	7	9.85E-02	1.61E-01
KEGG Orthology (KO) [BR:hsa00001]:09160 Human Diseases:09174 Infectious disease: parasitic	10	1.02E-01	1.61E-01
KEGG Orthology (KO) [BR:hsa00001]:09160 Human Diseases:09171 Infectious disease: bacterial:05133 Pertussis	5	1.31E-01	1.72E-01
Membrane trafficking [BR:hsa04131]:Endocytosis:Phagocytosis	5	1.31E-01	1.72E-01
KEGG Orthology (KO) [BR:hsa00001]:09160 Human Diseases:09161 Cancer: overview:05205 Proteoglycans in cancer	6	1.36E-01	1.72E-01
KEGG Orthology (KO) [BR:hsa00001]:09160 Human Diseases:09164 Neurodegenerative disease:05020 Prion disease	6	1.60E-01	1.88E-01

Exosome [BR:hsa04147]:Exosomal proteins:Exosomal proteins of other cancer cells	6	1.87E-01	1.88E-01
KEGG Orthology (KO) [BR:hsa00001]:09130 Environmental Information Processing:09133 Signaling molecules and interaction:04512 ECM-receptor interaction	6	1.87E-01	1.88E-01
KEGG Orthology (KO) [BR:hsa00001]:09160 Human Diseases:09172 Infectious disease: viral:05165 Human papillomavirus infection	5	1.88E-01	1.88E-01

M vs. C

KEGG hierarchical term	*count*	*p-value*	*q-value*
Peptidases and inhibitors [BR:hsa01002]:Peptidase inhibitors	18	9.06E-03	3.62E-02
Enzymes [BR:hsa01000]:3. Hydrolases:3.4 Acting on peptide bonds (peptidases):3.4.21 Serine endopeptidases	14	2.63E-02	5.27E-02
Peptidases and inhibitors [BR:hsa01002]:Serine peptidases:Family S1: chymotrypsin family	13	4.48E-02	5.34E-02
Exosome [BR:hsa04147]:Exosomal proteins:Exosomal proteins of other cancer cells	12	5.34E-02	5.34E-02

M vs. SC

KEGG hierarchical term	*count*	*p-value*	*q-value*
KEGG Orthology (KO) [BR:hsa00001]:09130 Environmental Information Processing:09133 Signaling molecules and interaction	21	1.06E-02	7.46E-02
Peptidases and inhibitors [BR:hsa01002]:Peptidase inhibitors	13	2.05E-02	7.46E-02
KEGG Orthology (KO) [BR:hsa00001]:09150 Organismal Systems:09151 Immune system:04610 Complement and coagulation cascades	20	2.27E-02	7.46E-02
KEGG Orthology (KO) [BR:hsa00001]:09160 Human Diseases:09167 Endocrine and metabolic disease	10	2.99E-02	7.46E-02
KEGG Orthology (KO) [BR:hsa00001]:09130 Environmental Information Processing:09132 Signal transduction:04151 PI3K-Akt signaling pathway	10	6.49E-02	1.30E-01
KEGG Orthology (KO) [BR:hsa00001]:09160 Human Diseases:09172 Infectious disease: viral:05165 Human papillomavirus infection	7	1.00E-01	1.34E-01
KEGG Orthology (KO) [BR:hsa00001]:09160 Human Diseases:09164 Neurodegenerative disease:05020 Prion disease	8	1.09E-01	1.34E-01
KEGG Orthology (KO) [BR:hsa00001]:09140 Cellular Processes:09142 Cell motility	7	1.27E-01	1.34E-01
KEGG Orthology (KO) [BR:hsa00001]:09140 Cellular Processes:09142 Cell motility:04810 Regulation of actin cytoskeleton	7	1.27E-01	1.34E-01
KEGG Orthology (KO) [BR:hsa00001]:09130 Environmental Information Processing:09133 Signaling molecules and interaction:04512 ECM-receptor interaction	8	1.34E-01	1.34E-01

S vs. C

KEGG hierarchical term	*count*	*p-value*	*q-value*
Peptidases and inhibitors [BR:hsa01002]:Serine peptidases	17	5.34E-04	3.93E-03

Peptidases and inhibitors [BR:hsa01002]:Serine peptidases:Family S1: chymotrypsin family	14	9.84E-04	3.93E-03
Enzymes [BR:hsa01000]:3. Hydrolases:3.4 Acting on peptide bonds (peptidases):3.4.21 Serine endopeptidases	14	1.61E-03	4.29E-03
KEGG Orthology (KO) [BR:hsa00001]:09150 Organismal Systems:09151 Immune system:04610 Complement and coagulation cascades	24	3.87E-03	7.73E-03
KEGG Orthology (KO) [BR:hsa00001]:09100 Metabolism:09107 Glycan biosynthesis and metabolism	8	1.44E-02	2.31E-02
Peptidases and inhibitors [BR:hsa01002]:Peptidase inhibitors	14	2.10E-02	2.62E-02
Peptidases and inhibitors [BR:hsa01002]:Peptidase inhibitors:Family I4: serpin family	8	2.29E-02	2.62E-02
Exosome [BR:hsa04147]:Exosomal proteins:Exosomal proteins of other cancer cells	9	1.07E-01	1.07E-01

M vs. S ∩ M vs. C ∩ M vs. SC

KEGG hierarchical term	*count*	*p-value*	*q-value*
KEGG Orthology (KO) [BR:hsa00001]:09130 Environmental Information Processing:09133 Signaling molecules and interaction:04080 Neuroactive ligand-receptor interaction	5	1.16E-03	3.58E-02
Membrane trafficking [BR:hsa04131]:Endocytosis:Phagocytosis:Opsonins	5	3.56E-03	4.60E-02
Peptidases and inhibitors [BR:hsa01002]:Peptidase inhibitors:Family I25: cystatin family	4	6.33E-03	4.60E-02
Peptidases and inhibitors [BR:hsa01002]:Peptidase inhibitors	9	6.86E-03	4.60E-02
KEGG Orthology (KO) [BR:hsa00001]:09130 Environmental Information Processing:09133 Signaling molecules and interaction	13	7.42E-03	4.60E-02
KEGG Orthology (KO) [BR:hsa00001]:09160 Human Diseases:09174 Infectious disease: parasitic:05142 Chagas disease	4	1.60E-02	8.29E-02
Membrane trafficking [BR:hsa04131]:Endocytosis:Phagocytosis	5	2.83E-02	1.17E-01
KEGG Orthology (KO) [BR:hsa00001]:09160 Human Diseases:09167 Endocrine and metabolic disease:04936 Alcoholic liver disease	4	3.20E-02	1.17E-01
KEGG Orthology (KO) [BR:hsa00001]:09160 Human Diseases:09167 Endocrine and metabolic disease	6	4.34E-02	1.17E-01
Membrane trafficking [BR:hsa04131]:Endocytosis	6	4.34E-02	1.17E-01
KEGG Orthology (KO) [BR:hsa00001]:09150 Organismal Systems:09151 Immune system:04610 Complement and coagulation cascades	11	4.66E-02	1.17E-01
Domain-containing proteins not elsewhere classified [BR:hsa04990]	8	4.89E-02	1.17E-01
KEGG Orthology (KO) [BR:hsa00001]:09180 Brite Hierarchies:09183 Protein families: signaling and cellular processes:04990 Domain-containing proteins not elsewhere classified [BR:hsa04990]	8	4.89E-02	1.17E-01
KEGG Orthology (KO) [BR:hsa00001]:09160 Human Diseases:09174 Infectious disease: parasitic	8	5.59E-02	1.24E-01
Glycosaminoglycan binding proteins [BR:hsa00536]:Heparan sulfate / Heparin	7	6.33E-02	1.28E-01
KEGG Orthology (KO) [BR:hsa00001]:09140 Cellular Processes:09141 Transport and catabolism:04145 Phagosome	5	6.61E-02	1.28E-01

KEGG Orthology (KO) [BR:hsa00001]:09160 Human Diseases:09171 Infectious disease: bacterial:05150 Staphylococcus aureus infection	6	7.11E-02	1.30E-01
KEGG Orthology (KO) [BR:hsa00001]:09160 Human Diseases:09164 Neurodegenerative disease:05020 Prion disease	5	9.22E-02	1.46E-01
KEGG Orthology (KO) [BR:hsa00001]:09160 Human Diseases:09171 Infectious disease: bacterial:05133 Pertussis	4	1.01E-01	1.46E-01
Exosome [BR:hsa04147]:Exosomal proteins:Exosomal proteins of other cancer cells	5	1.07E-01	1.46E-01
KEGG Orthology (KO) [BR:hsa00001]:09130 Environmental Information Processing:09133 Signaling molecules and interaction:04512 ECM-receptor interaction	5	1.07E-01	1.46E-01
KEGG Orthology (KO) [BR:hsa00001]:09180 Brite Hierarchies:09182 Protein families: genetic information processing:04131 Membrane trafficking [BR:hsa04131]	10	1.08E-01	1.46E-01
Membrane trafficking [BR:hsa04131]	10	1.08E-01	1.46E-01
KEGG Orthology (KO) [BR:hsa00001]:09160 Human Diseases:09172 Infectious disease: viral:05165 Human papillomavirus infection	4	1.40E-01	1.81E-01
KEGG Orthology (KO) [BR:hsa00001]:09140 Cellular Processes:09142 Cell motility	4	1.62E-01	1.89E-01
KEGG Orthology (KO) [BR:hsa00001]:09140 Cellular Processes:09142 Cell motility:04810 Regulation of actin cytoskeleton	4	1.62E-01	1.89E-01
Glycosaminoglycan binding proteins [BR:hsa00536]	7	1.72E-01	1.89E-01
KEGG Orthology (KO) [BR:hsa00001]:09180 Brite Hierarchies:09183 Protein families: signaling and cellular processes:00536 Glycosaminoglycan binding proteins [BR:hsa00536]	7	1.72E-01	1.89E-01
KEGG Orthology (KO) [BR:hsa00001]:09130 Environmental Information Processing:09132 Signal transduction:04151 PI3K-Akt signaling pathway	5	1.77E-01	1.89E-01
Exosome [BR:hsa04147]:Exosomal proteins:Exosomal proteins of bladder cancer cells	4	2.08E-01	2.08E-01
KEGG Orthology (KO) [BR:hsa00001]:09160 Human Diseases:09161 Cancer: overview:05205 Proteoglycans in cancer	4	2.08E-01	2.08E-01

M vs. S ∩ M vs. C ∩ S vs. C

KEGG hierarchical term	*count*	*p-value*	*q-value*
Peptidases and inhibitors [BR:hsa01002]:Peptidase inhibitors	5	9.35E-04	1.50E-02
KEGG Orthology (KO) [BR:hsa00001]:09150 Organismal Systems:09151 Immune system:04610 Complement and coagulation cascades	6	2.09E-03	1.67E-02
KEGG Orthology (KO) [BR:hsa00001]:09180 Brite Hierarchies:09181 Protein families: metabolism:01002 Peptidases and inhibitors [BR:hsa01002]	7	9.16E-03	3.66E-02
Peptidases and inhibitors [BR:hsa01002]	7	9.16E-03	3.66E-02
KEGG Orthology (KO) [BR:hsa00001]:09160 Human Diseases:09174 Infectious disease: parasitic	4	1.44E-02	4.60E-02
KEGG Orthology (KO) [BR:hsa00001]:09150 Organismal Systems:09151 Immune system	7	2.30E-02	6.12E-02
KEGG Orthology (KO) [BR:hsa00001]:09130 Environmental Information Processing:09132 Signal transduction	5	3.29E-02	6.30E-02

KEGG Orthology (KO) [BR:hsa00001]:09180 Brite Hierarchies:09181 Protein families: metabolism	7	3.59E-02	6.30E-02
Exosome [BR:hsa04147]	8	4.33E-02	6.30E-02
Exosome [BR:hsa04147]:Exosomal proteins	8	4.33E-02	6.30E-02
KEGG Orthology (KO) [BR:hsa00001]:09180 Brite Hierarchies:09183 Protein families: signaling and cellular processes:04147 Exosome [BR:hsa04147]	8	4.33E-02	6.30E-02
KEGG Orthology (KO) [BR:hsa00001]:09130 Environmental Information Processing:09133 Signaling molecules and interaction	4	5.61E-02	7.48E-02
KEGG Orthology (KO) [BR:hsa00001]:09140 Cellular Processes	5	1.06E-01	1.17E-01
KEGG Orthology (KO) [BR:hsa00001]:09160 Human Diseases:09161 Cancer: overview	4	1.06E-01	1.17E-01
KEGG Orthology (KO) [BR:hsa00001]:09130 Environmental Information Processing	5	1.17E-01	1.17E-01
KEGG Orthology (KO) [BR:hsa00001]:09150 Organismal Systems	8	1.17E-01	1.17E-01

M_{pair} vs. S_{pair}

KEGG hierarchical term	*count*	*p-value*	*q-value*
KEGG Orthology (KO) [BR:hsa00001]:09180 Brite Hierarchies:09181 Protein families: metabolism	8	5.55E-03	3.06E-02
Peptidases and inhibitors [BR:hsa01002]:Peptidase inhibitors	4	6.12E-03	3.06E-02
KEGG Orthology (KO) [BR:hsa00001]:09180 Brite Hierarchies:09183 Protein families: signaling and cellular processes	12	1.30E-02	4.25E-02
KEGG Orthology (KO) [BR:hsa00001]:09160 Human Diseases:09161 Cancer: overview	5	2.19E-02	4.25E-02
KEGG Orthology (KO) [BR:hsa00001]:09180 Brite Hierarchies:09181 Protein families: metabolism:01002 Peptidases and inhibitors [BR:hsa01002]	6	2.55E-02	4.25E-02
Peptidases and inhibitors [BR:hsa01002]	6	2.55E-02	4.25E-02
KEGG Orthology (KO) [BR:hsa00001]:09150 Organismal Systems:09151 Immune system:04610 Complement and coagulation cascades	4	4.45E-02	6.35E-02
KEGG Orthology (KO) [BR:hsa00001]:09140 Cellular Processes	5	8.18E-02	1.02E-01
KEGG Orthology (KO) [BR:hsa00001]:09160 Human Diseases	7	1.20E-01	1.33E-01
KEGG Orthology (KO) [BR:hsa00001]:09130 Environmental Information Processing	4	2.38E-01	2.38E-01
