# Supplementary material for: Acute and chronic blood serum proteome changes in patients with methanol poisoning
Source: Sci Rep. 2022 Dec 9;12:21379. doi: 10.1038/s41598-022-25492-9 (PMC9734099; doi:10.1038/s41598-022-25492-9)
Supplement: Supplementary file 8 — Supplementary Information 8. [file 41598_2022_25492_MOESM8_ESM.pdf]

Detection of potential N-terminal protein modifications originated from methanol

Methanol and its intermediate metabolites, like formaldehyde, are known to be responsible for protein structure modifications – especially N-terminal or side amino group modifications (Skrzydlewska, 2003). Non-enzymatic modifications of the N-terminal modifications of proteins with formaldehyde were at least confirmed in *in vitro* reactions (Ospina, Costin, Barry, & Vesper, 2011; Sykora et al., 2020; Yang et al., 2017) or blood samples of human patients in the smokers/nonsmokers comparison experiments ((Yang et al., 2017), modifications detected in samples from both groups). The common potential modifications known from non-enzymatic reactions of formaldehyde include N-terminal formation of the Schiff base, N-terminal amino acid formylation, methylation of the lysine ε-amino group, or creation of the imidazolidone group at the N-terminal valine (Ospina et al., 2011; Skrzydlewska, 2003; Tyihak, Trezl, & Rusznak, 1980). Due to search constraints and verification of the results, only N-terminal modifications were added to MaxQuant as possible modifications on the protein N-terminal and included in the search for the modified peptides. Of the 90 different N-terminal amino sequences found (106 including various acetylated sequences and sequences with different oxidation states of methionine), only 6 N-terminal peptides with a possible Schiff base were present, no peptides with a possible imidazolidone group on the N-terminal valine were present, and only 8 N-terminal peptides with potential formylation were identified. However, a closer analysis of the MaxQuant output has shown that the modified peptides in question often include several other potential modifications (methionine oxidation), miscleavage errors, and possibly methionine is also present at the beginning of the found modified peptides, which is not as common in mature proteins (Varland, Osberg, & Arnesen, 2015). Thus, these few cases of occurrence can, with high probability, also be considered a random identification by chance rather than a clear identification of the modified peptide (Burger, 2018; Nesvizhskii, 2010), even though they passed the threshold for successful identification. Formaldehyde is known to be endogenous in human blood (Heck, White, & Casanovaschmitz, 1982), and the imidazolidone group formation at the N-terminal valine of hemoglobin subunits was detected in a ratio of approximately 10^{-3} per unmodified molecules if ultraperformace liquid chromatography (UPLC) tandem mass spectrometry was used (Yang et al., 2017). To verify this potential modification, especially for hemoglobin, sequences for the α–, β–, and δ–subunits of hemoglobin, commonly present in blood, were downloaded from the UniProt database, and MaxQuant software was set to search only for hemoglobin molecules and its potential N-terminal modifications. Even with this limitation, MaxQuant did not detect proteins with the N-terminal valine imidazolidone group and found only unmodified peptides or peptides with N-terminal acetylation. This does not contradict the literature (Yang et al., 2017), where especially for this task, a targeted approach with a heavy-labeled standard and Selective Reaction Monitoring type of analysis and detection was used. The sensitivity of such methods is usually several orders of magnitude higher in comparison with our untargeted approach. Moreover, the mentioned study aimed at peptides originating from hemoglobin and red blood cells, which were unwanted contamination in our samples.

References

Burger, T. (2018). Gentle Introduction to the Statistical Foundations of False Discovery Rate in Quantitative Proteomics. *Journal of Proteome Research, 17*(1), 12-22. doi:10.1021/acs.jproteome.7b00170

Heck, H. D., White, E. L., & Casanovaschmitz, M. (1982). Determination of Formaldehyde in Biological Tissues by Gas-Chromatography Mass-Spectrometry. *Biomedical Mass Spectrometry, 9*(8), 347-353.

Nesvizhskii, A. I. (2010). A survey of computational methods and error rate estimation procedures for peptide and protein identification in shotgun proteomics. *Journal of Proteomics, 73*(11), 2092-2123. doi:10.1016/j.jprot.2010.08.009

Ospina, M., Costin, A., Barry, A. K., & Vesper, H. W. (2011). Characterization of N-terminal formaldehyde adducts to hemoglobin. *Rapid Communications in Mass Spectrometry, 25*(8), 1043-1050. doi:10.1002/rcm.4954

Skrzydlewska, E. (2003). Toxicological and metabolic consequences of methanol poisoning. *Toxicology Mechanisms and Methods, 13*(4), 277-293. doi:10.1080/713857189

Sykora, D., Jindrich, J., Kral, V., Jakubek, M., Tatar, A., Kejik, Z., . . . Zakharov, S. (2020). Formaldehyde Reacts with Amino Acids and Peptides with a Potential Role in Acute Methanol Intoxication. *Journal of Analytical Toxicology, 44*(8), 880-885. doi:10.1093/jat/bkaa039

Tyihak, E., Trezl, L., & Rusznak, I. (1980). Spontaneous N-Epsilon-Methylation of L-Lysine by Formaldehyde. *Pharmazie, 35*(1), 18-20.

Varland, S., Osberg, C., & Arnesen, T. (2015). N-terminal modifications of cellular proteins: The enzymes involved, their substrate specificities and biological effects. *Proteomics, 15*(14), 2385-2401. doi:10.1002/pmic.201400619

Yang, M., Ospina, M., Tse, C., Toth, S., Caudill, S. P., & Vesper, H. W. (2017). Ultraperformance Liquid Chromatography Tandem Mass Spectrometry Method To Determine Formaldehyde Hemoglobin Adducts in Humans as Biomarker for Formaldehyde Exposure. *Chemical Research in Toxicology, 30*(8), 1592-1598. doi:10.1021/acs.chemrestox.7b00114
