# Supplementary material for: Acute and chronic blood serum proteome changes in patients with methanol poisoning
Source: Sci Rep. 2022 Dec 9;12:21379. doi: 10.1038/s41598-022-25492-9 (PMC9734099; doi:10.1038/s41598-022-25492-9)
Supplement: Supplementary file 9 — Supplementary Information 9. [file 41598_2022_25492_MOESM9_ESM.pdf]

Identification of proteins with significant changes in M vs. S ∩ M vs. C ∩ S vs. C

The 15 proteins (**Table 4**), which are significant among all three analyzed groups (M vs. S, M vs. C, S vs. C), can be divided into four groups according to their functions – coagulation and plasminogen cascades, immune defense, stress-activated protein kinase pathway, and metabolism of vitamin A.

Note: proteins mentioned later in underlined style were detected in the full set of proteins with at least 50% occurrence in any group of M, S, and C (590 proteins). A significant increase or decrease, if not more specified, is related to the first set of the dual set comparison, i.e., M for M vs. S, S for S vs. C, etc. For triple comparison sets, a decrease or increase is related to M, i.e., for M vs. S ∩ M vs. C ∩ S vs. C or M vs. S ∩ M vs. C ∩ M vs. SC. A probable increase or decrease means 0.05 ≤ q-value < 0.15.

Blood coagulation cascade

Two main types of **kininogen** (decreased, detected as kininogen-1) are recognized – high-molecular-weight-kininogen (HMWK) and low-molecular-weight-kininogen (LMWK), originating from the alternative splicing of the same kininogen gene. HMWK is a multifunctional protein that serves as a cofactor in blood coagulation, fibrinolysis, and the complement system (Baird & Walsh, 2003). LMWK is, in contrast to HMWK, not directly involved in blood clotting, but only its by-products can be later converted and introduced to the coagulation pathway.

During the contact activation system, also known as the intrinsic pathway, the binding of HMWK, coagulation factor XII (probably increased in S vs. C), and prekallikrein (PK) to an anionic surface initiates blood coagulation and the kinin-kallikrein system through the activation of a cascade of enzymes (Schmaier, 2016). The blood clotting cascade consists of the intrinsic and extrinsic pathways, both of which create thrombin, a protease involved in blood clotting. The intrinsic pathway requires kininogen, specifically HMWK, as a cofactor. Both the intrinsic pathway, activated by a damaged surface, and its corresponding extrinsic pathway, activated when outside trauma activates tissue factor, culminate in the activation of a serine protease coagulation factor X (FX, increased in M vs. C, S vs. C, M vs. SC). FX is responsible for converting prothrombin (increased in M vs. C, S vs. C) into an important protease in clotting called thrombin, which itself participates in the clotting cascade by activating more enzymes and proteins downstream in the cascade. In the kinin-kallikrein system, the proteolytic cleavage of HMWK by the enzyme plasma kallikrein (increased in M vs. C, probably in S vs. C) makes bradykinin, an inflammatory mediator that can lower blood pressure by way of vasodilation but is also thought to cause localized edema, swelling, and pain (Schneider, Lumry, Vegh, Williams, & Schmalbach, 2007). HMWK and LMWK are also noncompetitive inhibitors of activated thrombin.

HMWK inhibits thrombin-stimulated **von Willebrand factor** (VWF, increased) release by human umbilical vein endothelial cells. Coagulation factor XI (FXI, probably increased in S vs. C) is able to restore the inhibited ability of thrombin to stimulate VWF release in the presence of low HMWK concentrations. Free HMWK or HMWK in complex with prekallikrein, but not in complex with FXI, probably interacts with the endothelium and can maintain endothelial cell quiescence by preventing endothelial stimulation by thrombin (Baird & Walsh, 2003). Platelet plug formation is dependent on VWF function. VWF plays an

essential role in primary hemostasis, bridging the binding of platelets to the subendothelium and other platelets, thereby promoting platelet plug formation in injured vessels (Thomazini et al., 2021). Coagulation factor VIII (decreased in M vs. S, M vs. SC, probably M vs. C) and VWF circulate in plasma in a tight non-covalent complex (Pegon et al., 2012).

Plasminogen cascade

Plasma kallikrein also mediates a *plasminogen cascade*. In the cascade, a serine protease plasmin, released from the plasminogen (decreased in M vs. S, M vs. C, M vs. SC, M_{pair} vs. S_{pair}), degrades fibrin blood clots and destroys coagulation factors (Selvarajan, Lund, Takeuchi, Craik, & Werb, 2001). It also cleaves fibrin, fibronectin, thrombospondin (decreased), laminin (probably increased, increased in M vs. S, M vs. SC), and von Willebrand factor. Plasmin is mainly derived from inactive plasminogen by tissue- or urokinase-type plasminogen activators (tPA or uPA) (Lin et al., 2020). Alcohol consumption is associated with a reduced risk for coronary heart disease, and this cardioprotection may be due, in part, to increased fibrinolysis. Plasmin is physiologically modulated by a specific inhibitor, **alpha-2-antiplasmin** (increased), and a nonspecific protease inactivator, alpha-2-macroglobulin (increased in S vs. C, probably increased in M vs. C). Plasminogen activation can also be downregulated by plasma **carboxypeptidase N** (CPN, increased) that cleaves C-terminal Lys residues on these plasminogen "receptors", which decreases its activation up to 1000-fold (Plow, Allampallam, & Redlitz, 1997; Redlitz, Tan, Eaton, & Plow, 1995). CPN also cleaves C-terminal Arg from bradykinin and contributes *in vivo* to the degradation of circulating kinins in blood plasma.
The plasminogen pathway is activated by protein C, which is known as autoprothrombin IIA or blood coagulation factor XIX. The activated form of protein C (APC) plays an important role in regulating anticoagulation, inflammatory processes, cell death, and maintaining the permeability of blood vessel walls. APC performs these processes primarily by proteolytically inactivating coagulation factor V_a and coagulation factor VIII_a. The generation of the APC is inhibited by **plasma serine protease inhibitor** (protein C inhibitor (PCI, SERPINA5), decreased), which is a heparin-dependent serine protease inhibitor. Serine proteases are controlled (inhibited) by SERPINs (serine protease inhibitors). Serpin peptidase inhibitor was found to be down-regulated in a study dealing with ethanol dosage and its influence on human fibroblast cells (Zamanian-Azodi, Rezaei-Tavirani, Rahmati-Rad, & Tavirani, 2016). PCI plays hemostatic roles in the blood plasma – it acts as an anticoagulant factor by inhibiting blood coagulation factors like prothrombin (increased in M vs. C and S vs. C), factor XI (probably increased in S vs. C), factor X_a (increased in M vs. C, S vs. C, M vs. SC), plasma kallikrein (increased in M vs. C, probably in S vs. C), and fibrinolytic enzymes such as tPA and uPA (Meijers et al., 1988). PCI also acts as a procoagulant and proinflammatory factor by inhibiting the anticoagulant APC and the generation of APC by the thrombin/thrombomodulin complex, as mentioned above.
Fibronectin (FN, increased) is a ubiquitous extracellular matrix glycoprotein that plays vital roles during tissue repair. The plasma form of FN circulates in the blood and, upon tissue injury, is incorporated into fibrin clots to exert effects on platelet function and to mediate hemostasis (To & Midwood, 2011). The N-terminal 70K region of fibronectin becomes cross-linked to fibrin clots by the formation of ε-(γ-glutamyl)-lysyl bonds catalyzed by thrombin-activated coagulation factor XIII_a (plasma transglutaminase, increased in M vs. C, M vs. SC, S vs. C, probably M vs. S, probably M_{pair} vs. S_{pair}) (Mosher, 1975).

Immune and inflammatory response

Monocyte differentiation antigen CD14 (CD14, increased) is a pattern recognition receptor on monocytes. This glycoprotein acts as a co-receptor for the detection of bacterial lipopolysaccharides and has membrane-bound (mCD14) and soluble (sCD14) forms (Kitchens, 2000; Tapping & Tobias, 2000). Liangpunsakul et al. (Liangpunsakul et al., 2017) found that the serum levels of sCD163 (increased in M vs. S and M vs. C) and sCD14 are significantly increased in excessive drinkers, notably in excessive drinkers with recent alcohol consumption. Excessive alcohol consumption could probably lead to the impairment of intestinal permeability and subsequent bacterial translocation from the gut into systemic circulation. Once in circulation, bacterial lipopolysaccharides can bind to the toll-like receptors on many cells, notably monocytes and macrophages, activating the inflammatory cytokines (Mogensen, 2009), which can be used as surrogates for the next monocyte activation. Monocytes and their activation play a crucial role in the first phase of atherogenesis (Hubacek & Poledne, 1999).

Complement factor I (increased) is a trypsin-like serine protease that plays an essential role in regulating the immune response by controlling all complement pathways. It inhibits these pathways by cleaving three peptide bonds in the alpha-chain of C3b and two bonds in C4b, thereby inactivating these proteins (Harrison & Lachmann, 1980). Its deficiency is strongly associated with recurrent bacterial infections.

JNK pathway, signaling

FYVE, RhoGEF, and PH domain-containing protein 6 (decreased) may activate cell division control protein 42 (Cdc42), a member of the Ras-like family (small GTPases) of Rho- and Rac proteins, by exchanging bound GDP for free GTP. It has been determined that Cdc42 actively assists in the progression of cancer. It is overexpressed in lung cancer, melanoma, colorectal adenocarcinoma, breast cancer, and testicular cancer. Elevated levels correlate with negative patient survival (Stengel & Zheng, 2011). Cdc42 activates the c-Jun N-terminal kinase (JNK, also called stress-activated protein kinase) pathway (JNK pathway), which is one of the major signaling components of the mitogen-activated protein kinase (MAPK) signaling pathway. It functions in the control of many cellular processes, including cytokine production, proliferation, embryonic development, and apoptosis. The JNK pathway controls the cell response to harmful extracellular stimuli such as inflammatory cytokines, UV-irradiation, gamma-irradiation, heat, oxidative stress, etc. These harmful stimuli may introduce DNA mutations or damage in the cells, and if this cannot be repaired, the cell is programmed for apoptosis (Keshet & Seger, 2010). The signaling to the JNK cascade often involves the Rho family GTPases Cdc42 and Rac. Those receptors or receptor-independent stress-induced membranal changes consequently transmit the signals to other proteins that can activate kinases in the MAP4K and sometimes the MAP3K levels of the JNK cascade (Keshet & Seger, 2010). The JNK cascade is also negatively regulated by **alpha-1-microglobulin** (bikunin, AMBP, increased) (Kobayashi, Suzuki, Hirashima, & Terao, 2003). This protein also interacts in chemical reactions and pathways, resulting in the breakdown of heme and its catabolism (Allhorn, Berggard, Nordberg, Olsson, & Akerstrom, 2002). The protein is believed to protect cells and tissues against the damage induced by abnormally high concentrations of free hemoglobin and/or reactive oxygen species (Olsson et al., 2012). AMBP also partially suppresses the immune response of lymphocytes and neutrophils (Akerstrom, Logdberg, Berggard, Osmark, & Lindqvist, 2000)

Vitamin A metabolism

Plasma retinol (precursor of retinal and retinoic acid (RA)) is bound by **retinol-binding protein** (RBP, increased) in blood. Vitamin A homeostasis requires the cellular retinol-binding protein for stimulation of the conversion of retinol into retinyl esters, serving as a storage form of vitamin A. However, ADH also facilitates the degradative metabolism of retinol into retinoic acid to protect against the toxic effects of high levels of vitamin A (Molotkov, Ghyselinck, Chambon, & Duester, 2004). Retinol-binding protein is then required to mobilize liver-stored vitamin A (Quadro et al., 1999). It has long been recognized that alcoholics are generally malnourished and can suffer from vitamin A deficiency, like a loss of night vision (nyctalopia) (Clugston & Blaner, 2012). It was found that ethanol inhibits cytosolic retinol dehydrogenase (RoDH) activity (transformation of retinol to retinal) but stimulates RA synthesis in a combination of microsomes and cytosol (Boerman & Napoli, 1996). Ethanol inhibition of cytosolic RoDH activity and stimulation of microsomal RoDH activity introduce possible alternative mechanisms for the impact of ethanol consumption on vitamin A metabolism and RA generation, unconnected to ADH isozymes (Napoli, 2000). Chronic excessive ethanol consumption decreases hepatic vitamin A (vitamin A = group of organic compounds that includes retinol, retinal, RA, and several provitamin A carotenoids) stores, and RBP is required to mobilize liver-stored vitamin A (Quadro et al., 1999). Serum carotene (vitamin A precursor) concentrations were also correlated to high-density lipoprotein (HDL) concentrations (where Apolipoprotein A I (increased in M vs. C, S vs. C) and Apolipoprotein A II (increased in M vs. C, probably increased in M vs. S, M vs. SC) are its most abundant components). Low concentrations of both were associated with increased cancer risk (Adams et al., 1985).

Apolipoprotein D (ApoD, increased) is a component of high-density lipoprotein (HDL), which has no marked similarity to other apolipoprotein sequences. It shows a high degree of homology to plasma retinol-binding protein and other members of the alpha-2-microglobulin protein superfamily of carrier proteins (lipocalins). ApoD is closely associated with the enzyme lecithin: cholesterol acyltransferase – an enzyme involved in lipoprotein metabolism (Perdomo & Dong, 2009). It could be a multi-ligand, multi-function protein. RA induces ApoD. Lopez-Boado et al. (LopezBoado, Klaus, Dawson, & LopezOtin, 1996) present evidence of the involvement of a retinoic-acid-receptor-dependent signaling pathway in the stimulation of ApoD gene expression by retinoids in breast cancer cells. The activation of this cascade leads to significant inhibition of cell proliferation in breast cancer. In another study (Dassati, Waldner, & Schweigreiter, 2014), these results indicate the upregulation of ApoD is part of an evolutionarily conserved stress response pathway in the aging brain. By its antioxidant and anti-inflammatory activity, ApoD protects against the age-associated oxidative stress that builds up because of increasing reactive oxygen species levels. ApoD probably achieves these neuroprotective effects by reducing lipid peroxidation.

Already mentioned plasma serine protease inhibitor (PCI) acting in body fluids and secretions (Laurell, Christensson, Abrahamsson, Stenflo, & Lilja, 1992) in a plasminogen cascade may also play a non-inhibitory role in seminal plasma and urine as a hydrophobic hormone carrier by its binding to RA (Jerabek, Zechmeister-Machhart, Binder, & Geiger, 2001). This hypothesis is also supported by the fact that PCI is abundantly expressed in organs and tissues requiring retinoic acid for maturation and differentiation processes (Laurell et al., 1992; Prendes et al., 1999). Retinoic acid, the transcriptionally active metabolite of vitamin A, has

long been recognized as a regulator of neuronal patterning in the developing embryo and an inducer of molecular events that lead to the development of a fully differentiated neuron (McCaffery, Zhang, & Crandall, 2006). The activity of PCI is higher when the protein is biotinylated (Prendes et al., 1999). The other found protein, **biotinidase** (increased), catalyzes a release of biotin from biocytin, the product of biotin-dependent carboxylase degradation.

References

Adams, L. L., LaPorte, R. E., Watkins, L. O., Savage, D. D., Bates, M., D'Antonio, J. A., & Kuller, L. H. (1985). The association of lipoprotein cholesterol with vitamin A. *Cancer, 56*(11), 2593-2597. doi:10.1002/1097-0142(19851201)56:11<2593::aid-cncr2820561110>3.0.co;2-8

Akerstrom, B., Logdberg, L., Berggard, T., Osmark, P., & Lindqvist, A. (2000). alpha(1)-Microglobulin: a yellow-brown lipocalin. *Biochim Biophys Acta, 1482*(1-2), 172-184. doi:10.1016/s0167-4838(00)00157-6

Allhorn, M., Berggard, T., Nordberg, J., Olsson, M. L., & Akerstrom, B. (2002). Processing of the lipocalin alpha(1)-microglobulin by hemoglobin induces heme-binding and heme-degradation properties. *Blood, 99*(6), 1894-1901. doi:10.1182/blood.V99.6.1894

Baird, T. R., & Walsh, P. N. (2003). Factor XI, but not prekallikrein, blocks high molecular weight kininogen binding to human umbilical vein endothelial cells. *Journal of Biological Chemistry, 278*(23), 20618-20623. doi:10.1074/jbc.M300224200

Boerman, M. H., & Napoli, J. L. (1996). Cellular retinol-binding protein-supported retinoic acid synthesis. Relative roles of microsomes and cytosol. *Journal of Biological Chemistry, 271*(10), 5610-5616. doi:10.1074/jbc.271.10.5610

Clugston, R. D., & Blaner, W. S. (2012). The Adverse Effects of Alcohol on Vitamin A Metabolism. *Nutrients, 4*(5), 356-371. doi:10.3390/nu4050356

Dassati, S., Waldner, A., & Schweigreiter, R. (2014). Apolipoprotein D takes center stage in the stress response of the aging and degenerative brain. *Neurobiology of Aging, 35*(7), 1632-1642. doi:10.1016/j.neurobiolaging.2014.01.148

Harrison, R. A., & Lachmann, P. J. (1980). The physiological breakdown of the third component of human complement. *Mol Immunol, 17*(1), 9-20. doi:10.1016/0161-5890(80)90119-4

Hubacek, J. A., & Poledne, R. (1999). The common cDNA and amino acid sequences of the CD14 (myeloid cell-specific leucine-rich glycoprotein) receptor. *Physiological Research, 48*(4), 323-326. Retrieved from <Go to ISI>://WOS:000083166500012

Jerabek, I., Zechmeister-Machhart, M., Binder, B. R., & Geiger, M. (2001). Binding of retinoic acid by the inhibitory serpin protein C inhibitor. *European Journal of Biochemistry, 268*(22), 5989-5996. doi:DOI 10.1046/j.0014-2956.2001.02560.x

Keshet, Y., & Seger, R. (2010). The MAP Kinase Signaling Cascades: A System of Hundreds of Components Regulates a Diverse Array of Physiological Functions. *Map Kinase Signaling Protocols, Second Edition, 661*, 3-38. doi:10.1007/978-1-60761-795-2_1

Kitchens, R. L. (2000). Role of CD14 in cellular recognition of bacterial lipopolysaccharides. *Cd14 in the Inflammatory Response, 74*, 61-82. Retrieved from <Go to ISI>://WOS:000171059200005

Kobayashi, H., Suzuki, M., Hirashima, Y., & Terao, T. (2003). The protease inhibitor bikunin, a novel anti-metastatic agent. *Biological Chemistry, 384*(5), 749-754. doi:Doi 10.1515/Bc.2003.083

Laurell, M., Christensson, A., Abrahamsson, P. A., Stenflo, J., & Lilja, H. (1992). Protein-C Inhibitor in Human-Body Fluids - Seminal Plasma Is Rich in Inhibitor Antigen

Deriving from Cells Throughout the Male Reproductive-System. *Journal of Clinical Investigation, 89*(4), 1094-1101. doi:Doi 10.1172/Jci115689

Liangpunsakul, S., Toh, E., Ross, R. A., Heathers, L. E., Chandler, K., Oshodi, A., . . . Nelson, D. E. (2017). Quantity of alcohol drinking positively correlates with serum levels of endotoxin and markers of monocyte activation. *Scientific Reports, 7*. doi:10.1038/s41598-017-04669-7

Lin, H. L., Xu, L. N., Yu, S. J., Hong, W. J., Huang, M. D., & Xu, P. (2020). Therapeutics targeting the fibrinolytic system. *Experimental and Molecular Medicine, 52*(3), 367-379. doi:10.1038/s12276-020-0397-x

LopezBoado, Y. S., Klaus, M., Dawson, M. I., & LopezOtin, C. (1996). Retinoic acid-induced expression of apolipoprotein D and concomitant growth arrest in human breast cancer cells are mediated through a retinoic acid receptor RAR alpha-dependent signaling pathway. *Journal of Biological Chemistry, 271*(50), 32105-32111. doi:DOI 10.1074/jbc.271.50.32105

McCaffery, P., Zhang, J. H., & Crandall, J. E. (2006). Retinoic acid signaling and function in the adult hippocampus. *Journal of Neurobiology, 66*(7), 780-791. doi:10.1002/neu.20237

Meijers, J. C. M., Kanters, D. H. A. J., Vlooswijk, R. A. A., Vanerp, H. E., Hessing, M., & Bouma, B. N. (1988). Inactivation of Human-Plasma Kallikrein and Factor-Xia by Protein-C Inhibitor. *Biochemistry, 27*(12), 4231-4237. doi:DOI 10.1021/bi00412a005

Mogensen, T. H. (2009). Pathogen Recognition and Inflammatory Signaling in Innate Immune Defenses. *Clinical Microbiology Reviews, 22*(2), 240-+. doi:10.1128/Cmr.00046-08

Molotkov, A., Ghyselinck, N. B., Chambon, P., & Duester, G. (2004). Opposing actions of cellular retinol-binding protein and alcohol dehydrogenase control the balance between retinol storage and degradation. *Biochemical Journal, 383*, 295-302. doi:10.1042/Bj20040621

Mosher, D. F. (1975). Cross-Linking of Cold-Insoluble Globulin by Fibrin-Stabilizing Factor. *Journal of Biological Chemistry, 250*(16), 6614-6621. Retrieved from <Go to ISI>://WOS:A1975AN94400064

Napoli, J. L. (2000). Retinoic acid: Its biosynthesis and metabolism. *Progress in Nucleic Acid Research and Molecular Biology, Vol 63, 63*, 139-188. doi:Doi 10.1016/S0079-6603(08)60722-9

Olsson, M. G., Allhorn, M., Bulow, L., Hansson, S. R., Ley, D., Olsson, M. L., . . . Akerstrom, B. (2012). Pathological Conditions Involving Extracellular Hemoglobin: Molecular Mechanisms, Clinical Significance, and Novel Therapeutic Opportunities for alpha(1)-Microglobulin. *Antioxidants & Redox Signaling, 17*(5), 813-846. doi:10.1089/ars.2011.4282

Pegon, J. N., Kurdi, M., Casari, C., Odouard, S., Denis, C. V., Christophe, O. D., & Lenting, P. J. (2012). Factor VIII and von Willebrand factor are ligands for the carbohydrate-receptor Siglec-5. *Haematologica-the Hematology Journal, 97*(12), 1855-1863. doi:10.3324/haematol.2012.063297

Perdomo, G., & Dong, H. H. (2009). Apolipoprotein D in lipid metabolism and its functional implication in atherosclerosis and aging. *Aging-Us, 1*(1), 17-27. Retrieved from <Go to ISI>://WOS:000276347200005

Plow, E. F., Allampallam, K., & Redlitz, A. (1997). The plasma carboxypeptidases and the regulation of the plasminogen system. *Trends in Cardiovascular Medicine, 7*(3), 71-75. doi:Doi 10.1016/S1050-1738(97)00012-1

Prendes, M. J., Bielek, E., Zechmeister-Machhart, M., Vanyek-Zavadil, E., Carroll, V. A., Breuss, J., . . . Geiger, M. (1999). Synthesis and ultrastructural localization of protein

C inhibitor in human platelets and megakaryocytes. *Blood, 94*(4), 1300-1312. doi:DOI 10.1182/blood.V94.4.1300.416k26_1300_1312

Quadro, L., Blaner, W. S., Salchow, D. J., Vogel, S., Piantedosi, R., Gouras, P., . . . Gottesman, M. E. (1999). Impaired retinal function and vitamin A availability in mice lacking retinol-binding protein. *Embo Journal, 18*(17), 4633-4644. doi:DOI 10.1093/emboj/18.17.4633

Redlitz, A., Tan, A. K., Eaton, D. L., & Plow, E. F. (1995). Plasma Carboxypeptidases as Regulators of the Plasminogen System. *Journal of Clinical Investigation, 96*(5), 2534-2538. doi:Doi 10.1172/Jci118315

Selvarajan, S., Lund, L. R., Takeuchi, T., Craik, C. S., & Werb, Z. (2001). A plasma kallikrein-dependent plasminogen cascade required for adipocyte differentiation. *Nature Cell Biology, 3*(3), 267-275. doi:Doi 10.1038/35060059

Schmaier, A. H. (2016). The contact activation and kallikrein/kinin systems: pathophysiologic and physiologic activities. *Journal of Thrombosis and Haemostasis, 14*(1), 28-39. doi:10.1111/jth.13194

Schneider, L., Lumry, W., Vegh, A., Williams, A. H., & Schmalbach, T. (2007). Critical role of kallikrein in hereditary angioedema pathogenesis: A clinical trial of ecallantide, a novel kallikrein inhibitor. *Journal of Allergy and Clinical Immunology, 120*(2), 416-422. doi:10.1016/j.jaci.2007.04.028

Stengel, K., & Zheng, Y. (2011). Cdc42 in oncogenic transformation, invasion, and tumorigenesis. *Cellular Signalling, 23*(9), 1415-1423. doi:10.1016/j.cellsig.2011.04.001

Tapping, R. I., & Tobias, P. S. (2000). Soluble CD14-mediated cellular responses to lipopolysaccharide. *Cd14 in the Inflammatory Response, 74*, 108-121. Retrieved from <Go to ISI>://WOS:000171059200007

Thomazini, C. M., Sachetto, A. T. A., de Albuquerque, C. Z., Mattaraia, V. G. D., de Oliveira, A. K., Serrano, S. M. D., . . . Santoro, M. L. (2021). Involvement of von Willebrand factor and botrocetin in the thrombocytopenia induced by Bothrops jararaca snake venom. *Plos Neglected Tropical Diseases, 15*(9). doi:10.1371/journal.pntd.0009715

To, W. S., & Midwood, K. S. (2011). Plasma and cellular fibronectin: distinct and independent functions during tissue repair. *Fibrogenesis & Tissue Repair, 4*. doi:10.1186/1755-1536-4-21

Zamanian-Azodi, M., Rezaei-Tavirani, M., Rahmati-Rad, S., & Tavirani, M. R. (2016). Ethanol and Cancer Induce Similar Changes on Protein Expression Pattern of Human Fibroblast Cell. *Iranian Journal of Pharmaceutical Research, 15*, 175-184. Retrieved from <Go to ISI>://WOS:000372435700018
