# Supplementary material for: Acute and chronic blood serum proteome changes in patients with methanol poisoning
Source: Sci Rep. 2022 Dec 9;12:21379. doi: 10.1038/s41598-022-25492-9 (PMC9734099; doi:10.1038/s41598-022-25492-9)
Supplement: Supplementary file 10 — Supplementary Information 10. [file 41598_2022_25492_MOESM10_ESM.pdf]

Identification of proteins with significant changes in M vs. S ∩ M vs. C ∩ M vs. SC, and pairwise in M_{pair} vs. S_{pair}

Note: proteins mentioned later in underlined style were detected in the full set of proteins with at least 50% occurrence in any group of M, S, and C (590 proteins). A significant increase or decrease, if not more specified, is related to the first set of the dual set comparison, i.e., M for M vs. S, S for S vs. C, etc. For triple comparison sets, a decrease or increase is related to M, i.e., for M vs. S ∩ M vs. C ∩ S vs. C or M vs. S ∩ M vs. C ∩ M vs. SC. A probable increase or decrease means $0.05 \leq$ q-value < 0.15.

The protein with the most significant change in this comparison is **inter-alpha-trypsin inhibitor heavy chain H4** (ITIH4, decreased), involved in inflammatory responses to trauma and also in coagulation and plasminogen cascades. It may also play a role in liver regeneration and development. ITIH4 is involved in a family of structurally related serine protease inhibitors (IaIp) found in relatively high concentrations in human plasma. It is implicated to have a role for IaIp in sepsis and have the potential to be a biomarker in sepsis and cancer (Fries & Kaczmarczyk, 2003; Lim et al., 2003; Salier, Rouet, Raguenez, & Daveau, 1996). In blood, ITIH4 is cleaved by kallikrein into two smaller forms. The light chain, bikunin (AMBP), inhibits several serine proteases, such as trypsin, plasmin, human leukocyte elastase, cathepsin G, and coagulation factor X_a (Fries & Kaczmarczyk, 2003; Salier et al., 1996). The decreased level of ITIH4 in our study led to an increase in blood coagulation. During infections, the level of ITIH4 is increased (Nakamura et al., 2019; Pineiro et al., 2004). **Plasminogen** (decreased) dissolves the fibrin of blood clots and acts as a proteolytic factor in various other processes, including embryonic development, tissue remodeling, tumor invasion, and inflammation. It activates the urokinase-type plasminogen activator, different collagenases, and several complement zymogens, e.g., Complement C1 and C5. The breakdown of fibronectin and laminin leads to cell detachment and apoptosis (Rossignol et al., 2004). **Heparanase** (HS, decreased) cleaves heparan sulfate proteoglycans into heparan sulfate side chains and core proteoglycans, which are the main components of the extracellular matrix (Sarrazin, Lamanna, & Esko, 2011). HS improves the production of coagulation factor Xa and subsequently activates the coagulation system by up-regulating the expression of the blood-tissue factor clotting initiator and directly increasing its activity. HS simultaneously performs other non-enzymatic functions, promoting the release and diffusion of various molecules associated with HS, such as growth factors, cytokines, and enzymes. It has been shown to have the ability to trigger various signaling pathways by interacting with transmembrane proteins (Masola, Bellin, Gambaro, & Onisto, 2018). **Carboxypeptidase Q** (decreased) belongs to carboxypeptidases with function in blood clotting, growth factor production, wound healing, reproduction, and many other processes. Glycoprotein **thrombospondin** (decreased) potentiates platelet and protein interactions, may take part in cellular adhesion and binds to complex carbohydrates such as heparin (Austen, 1994). It can be involved in several aspects of blood coagulation. When secreted from platelets, it binds to their surface and may be involved in platelet aggregation, presumably by stabilizing fibrinogen binding to platelets. This protein is also expressed in various processes, including cell proliferation, the inflammatory response to central nervous system damage, the regulation of vascular inflammation, the adaptive response of the heart to pressure overload, and

myocardial function and remodeling. It binds to the structural proteins of the extracellular matrix and modulates them in response to tissue damage (Bale & Mosher, 1986).

Regarding immune and inflammation processes, **complement C3** (C3, increased) plays a central role in activating the complement system (a part of the immune system). Its processing by C3 convertase is the main reaction in both classical and alternative complement cascades. **Collectins** (increased) are secreted proteins that play important roles in the innate immune system by binding to carbohydrate antigens on microorganisms, facilitating their recognition and removal (Nauser, Howard, Fanelli, Farrar, & Sacks, 2018). **Alpha-2-HS-glycoprotein** (decreased) probably has a role in the organism's defense mechanism. Its serum level is decreased in trauma patients (Lebreton et al., 1979). Besides acting as a negative acute phase reactant, its role could include opsonic functions or enhancing the phagocytic function of human monocytes. Abiodun et al. (Abiodun, Ihongbe, & Dati, 1985) confirmed that serum levels of alpha-2-HS-glycoprotein decrease in children with protein-energy malnutrition without bacterial infections. **Beta-2-glycoprotein** (β2GPI, decreased), together with C-reactive protein and thrombomodulin, possess the dual capability of up- and downregulating the complement and coagulation systems depending upon the external stimulus. This protein is also capable of adopting at least two distinct structural forms, with a unique five-domain protein comprising four similar complement control protein (CCP)-like domains (DI to DIV) and one different domain (DV) with a large lysine loop (1C1Z). β2GPI is a protein with a key role in hemostasis, homeostasis, and immunity. Although β2GPI has several proposed roles in the coagulation and complement cascades, they have been incompletely defined (McDonnell et al., 2020). **Serglycin** (decreased) has been initially characterized as an intracellular proteoglycan expressed by hematopoietic cells. This protein is mainly expressed during inflammation. All inflammatory cells synthesize serglycin and store it in granules, where it interacts with numerous inflammatory mediators, such as proteases, chemokines, cytokines, and growth factors. Serglycin is implicated in their storage in the granules and their protection since they are secreted as complexes and delivered to their targets after secretion (Korpetinou et al., 2014). **Titin** (decreased) is part of the sarcomere and interacts with the myosin and actin filaments (Herzog, 2018). However, titin has been associated with various functions, including forming cytotoxic amyloid aggregates (Smirnova et al., 2021). These formations activate inflammatory processes and the development of neurodegenerative diseases in humans by activating a complement system. The activation is triggered by binding two components of the complement system C1q and C3b and titin amyloid aggregates (Bobylev et al., 2021). **Bifunctional epoxide hydrolase 2** (decreased) is a bifunctional enzyme with two catalytic domains: a C-terminal epoxide hydrolase domain and an N-terminal phosphatase domain. The C-terminal epoxide hydrolase domain has variable cardiovascular and anti-inflammatory effects (EnayetAllah et al., 2008).

The protein FYVE of the JNK pathway mentioned in the previous chapter was also found, as well as proteins that could not be assigned to any group. Thus, **gelsolin** (decreased) is the most expressed member of the actin-separating protein family and is associated with cell survival, membrane transport, cytokinesis, and phagocytosis. In addition to muscle tissue, gelsolin is expressed in the human central nervous system (Kwiatkowski, 1988). Gelsolin regulates the architecture and dynamics of cells by capping, severing, and nucleating actin filaments. Its abnormal expression has been reported in many types of tumors. The frequent

silencing of gelsolin expression in various cancers has been reported (Kim et al., 2004; Noske et al., 2005).

Other proteins regarding cell cycle and developmental processes were also found. **Angiogenin** (decreased) binds to actin on endothelial cells and the surface of normal vascularization and malignant tissues. The initiation of angiogenesis occurs after the binding of angiogenin to actin located on endothelial cell membranes and the subsequent activation of the plasminogen activator, which in turn leads to plasmin formation. Plasmin, in combination with several activated matrix metalloproteinases, destroys the laminin (probably increased, increased in M vs. S, M vs. SC) and fibronectin of the basement membrane. This effect is necessary for endothelial cell migration during neovascularization (Yurina et al., 2021). **Fibroblast growth factor receptors** (decreased) play a crucial role in both developmental and adult cells. This protein is also implicated in many cancers (urothelial carcinoma, ovarian cancer, hepatocellular carcinoma, and adenocarcinoma) (Dai, Zhou, Chen, Xu, & Chen, 2019). **Semaphorins** (increased) control synaptogenesis, the density and maturation of dendritic spines, and axon pruning. They also inhibit axonal extension by providing local signals to specify territories inaccessible for growing axons (Pasterkamp & Giger, 2009). Due to their function in regulating synaptic physiology and neuronal excitability in the mature hippocampus, semaphorins are also implicated in several developmental, psychiatric, and neurodegenerative disorders. However, how semaphorins influence neuronal structure during adult nervous system homeostasis or following injury and disease is unclear (Carulli, de Winter, & Verhaagen, 2021).

Proteins with significant changes only in M vs. S

Among the first 15 proteins with the most significant changes between M and S, almost all the proteins except three are also in M vs. S ∩ M vs. C ∩ M vs. SC and were mentioned earlier. The remaining ones are protocadherin beta-12, serglycin, and interleukin-1 receptor accessory protein. All three are significantly decreased in M vs. S. and M vs. SC, but they are all increased in S vs. C (sometimes even significantly). Close values for protein intensity quantification values in samples from M and C disqualify them from being significantly changed in M vs. S ∩ M vs. C ∩ M vs. SC.

While cell adhesion protein **protocadherin beta-12** (decreased in M vs. S, M vs. SC, probably decreased in M_{pair} vs. S_{pair}, increased in S vs. C), as other protocadherins, may be involved in the establishment and maintenance of specific neuronal connections in the brain (Morishita & Yagi, 2007), the levels of other cadherins, even protocadherin-12, are not significantly changed.

Serglycin (decreased in M vs. S, M vs. SC, M_{pair} vs. S_{pair}, probably decreased in M vs. C, increased in S vs. C) is a low molecular glycoprotein expressed in hematopoietic cells, endothelial cells, and macrophages. It plays a role in cytotoxic cell granule-mediated apoptosis by forming a complex with granzyme B, which is delivered to cells by perforin to induce apoptosis. It regulates the secretion of tumor necrosis factor alpha and may also regulate protease secretion.

The **interleukin-1 receptor accessory protein** (decreased in M vs. S, M vs. SC, probably decreased in M_{pair} vs. S_{pair}), an associate protein for interleukin-1 receptor, is an important part

of the interleukin-1 beta system (IL-1β) – a prominent pro-inflammatory pathway responsible for the initiation and regulation of immune responses. In particular, its main cytokine ligand, interleukin-1-beta, has emerged as a key regulator of the ethanol-induced neuroimmune response, contributing to ethanol drinking and the development of ethanol dependence. Although essential for resistance to infections, IL-1β also exacerbates damage during chronic disease and acute tissue injuries (Dinarello, 2010; Lopez-Castejon & Brough, 2011). Preclinical studies using mice animal models of ethanol drinking and dependence provide strong support for the link between the IL-1β system and acute and chronic ethanol-induced changes in the brain – the alterations in the interleukin system change the ethanol preference and drinking, ethanol-induced sedation, and withdrawal severity (Patel et al., 2019). Mice with a knockout of the interleukin-1 receptor gene showed reduced sensitivity to the sedative effects of ethanol and flurazepam and the increased severity of acute ethanol withdrawal (Blednov, Benavidez, Black, Mayfield, & Harris, 2015). The methanol intoxication and treatment experiments on a rat optic nerve model showed an increased expression of IL-1β (Tasli et al., 2018). For other found interleukins, like interleukin-6 receptor subunit beta (increased in M vs. C, probably increased in M vs. C), a decrease in response to moderate ethanol consumption is also known from the literature (Huang et al., 2017).

References

Abiodun, P. O., Ihongbe, J. C., & Dati, F. (1985). Decreased Levels of Alpha2-Hs-Glycoprotein in Children with Protein-Energy-Malnutrition. *European Journal of Pediatrics, 144*(4), 368-369. doi:Doi 10.1007/Bf00441779

Austen, D. E. G. (1994). 29 - Clinical Biochemistry of Blood Coagulation. In D. L. Williams & V. Marks (Eds.), *Scientific Foundations of Biochemistry in Clinical Practice (Second Edition)* (pp. 495-513): Butterworth-Heinemann.

Bale, M. D., & Mosher, D. F. (1986). THROMBOSPONDIN IS A SUBSTRATE FOR BLOOD-COAGULATION FACTOR-XIIIA. *Biochemistry, 25*(19), 5667-5673. doi:10.1021/bi00367a048

Blednov, Y. A., Benavidez, J. M., Black, M., Mayfield, J., & Harris, R. A. (2015). Role of interleukin-1 receptor signaling in the behavioral effects of ethanol and benzodiazepines. *Neuropharmacology, 95*, 309-320. doi:10.1016/j.neuropharm.2015.03.015

Bobylev, A. G., Fadeev, R. S., Bobyleva, L. G., Kobyakova, M. I., Shlyapnikov, Y. M., Popov, D. V., & Vikhlyantsev, I. M. (2021). Amyloid Aggregates of Smooth-Muscle Titin Impair Cell Adhesion. *International journal of molecular sciences, 22*(9). doi:10.3390/ijms22094579

Carulli, D., de Winter, F., & Verhaagen, J. (2021). Semaphorins in Adult Nervous System Plasticity and Disease. *Frontiers in Synaptic Neuroscience, 13*. doi:10.3389/fnsyn.2021.672891

Dai, S., Zhou, Z., Chen, Z., Xu, G., & Chen, Y. (2019). Fibroblast Growth Factor Receptors (FGFRs): Structures and Small Molecule Inhibitors. *Cells, 8*(6), 614. doi:10.3390/cells8060614

Dinarello, C. A. (2010). Anti-inflammatory Agents: Present and Future. *Cell, 140*(6), 935-950. doi:10.1016/j.cell.2010.02.043

EnayetAllah, A. E., Luria, A., Luo, B., Tsai, H. J., Sura, P., Hammock, B. D., & Grant, D. F. (2008). Opposite regulation of cholesterol levels by the phosphatase and hydrolase domains of soluble epoxide hydrolase. *J Biol Chem, 283*(52), 36592-36598. doi:10.1074/jbc.M806315200

Fries, E., & Kaczmarczyk, A. (2003). Inter-alpha-inhibitor, hyaluronan and inflammation. *Acta Biochimica Polonica, 50*(3), 735-742. doi:035003735

Herzog, W. (2018). The multiple roles of titin in muscle contraction and force production. *Biophysical reviews, 10*(4), 1187-1199. doi:10.1007/s12551-017-0395-y

Huang, Y. Y., Li, Y. M., Zheng, S. C., Yang, X., Wang, T. H., & Zeng, J. (2017). Moderate alcohol consumption and atherosclerosis Meta-analysis of effects on lipids and inflammation. *Wiener Klinische Wochenschrift, 129*(21-22), 835-843. doi:10.1007/s00508-017-1235-6

Kim, J. H., Choi, Y. K., Kwon, H. J., Yang, H. K., Choi, J. H., & Kim, D. Y. (2004). Downregulation of gelsolin and retinoic acid receptor beta expression in gastric cancer tissues through histone deacetylase 1. *Journal of Gastroenterology and Hepatology, 19*(2), 218-224. doi:DOI 10.1111/j.1440-1746.2004.03336.x

Korpetinou, A., Skandalis, S., Labropoulou, V., Smirlaki, G., Noulas, A., Karamanos, N., & THEOCHARIS, A. (2014). Serglycin: At the Crossroad of Inflammation and Malignancy. *Frontiers in Oncology, 3*. doi:10.3389/fonc.2013.00327

Kwiatkowski, D. J. (1988). Predominant Induction of Gelsolin and Actin-Binding Protein during Myeloid Differentiation. *Journal of Biological Chemistry, 263*(27), 13857-13862. doi:10.1016/S0021-9258(18)68322-X

Lebreton, J. P., Joisel, F., Raoult, J. P., Lannuzel, B., Rogez, J. P., & Humbert, G. (1979). Serum Concentration of Human Alpha2 Hs Glycoprotein during the Inflammatory Process - Evidence That Alpha2 Hs Glycoprotein Is a Negative Acute-Phase Reactant. *Journal of Clinical Investigation, 64*(4), 1118-1129. doi:Doi 10.1172/Jci109551

Lim, Y. P., Bendelja, K., Opal, S. M., Siryaporn, E., Hixson, D. C., & Palardy, J. E. (2003). Correlation between mortality and the levels of inter-alpha inhibitors in the plasma of patients with severe sepsis. *Journal of Infectious Diseases, 188*(6), 919-926. doi:Doi 10.1086/377642

Lopez-Castejon, G., & Brough, D. (2011). Understanding the mechanism of IL-1 beta secretion. *Cytokine & Growth Factor Reviews, 22*(4), 189-195. doi:10.1016/j.cytogfr.2011.10.001

Masola, V., Bellin, G., Gambaro, G., & Onisto, M. (2018). Heparanase: A Multitasking Protein Involved in Extracellular Matrix (ECM) Remodeling and Intracellular Events. *Cells, 7*(12), 236. doi:10.3390/cells7120236

McDonnell, T., Wincup, C., Buchholz, I., Pericleous, C., Giles, I., Ripoll, V., . . . Rahman, A. (2020). The role of beta-2-glycoprotein I in health and disease associating structure with function: More than just APS. *Blood Reviews, 39*, 14. doi:10.1016/j.blre.2019.100610

Morishita, H., & Yagi, T. (2007). Protocadherin family: diversity, structure, and function. *Current Opinion in Cell Biology, 19*(5), 584-592. doi:10.1016/j.ceb.2007.09.006

Nakamura, N., Hatano, E., Iguchi, K., Sato, M., Kawaguchi, H., Ohtsu, I., . . . Ikegawa, M. (2019). Elevated levels of circulating ITIH4 are associated with hepatocellular carcinoma with nonalcoholic fatty liver disease: from pig model to human study. *Bmc Cancer, 19*. doi:10.1186/s12885-019-5825-8

Nauser, C. L., Howard, M. C., Fanelli, G., Farrar, C. A., & Sacks, S. (2018). Collectin-11 (CL-11) Is a Major Sentinel at Epithelial Surfaces and Key Pattern Recognition Molecule in Complement-Mediated Ischaemic Injury. *Frontiers in Immunology, 9*. doi:10.3389/fimmu.2018.02023

Noske, A., Denkert, C., Schober, H., Sers, C., Zhumabayeva, B., Weichert, W., . . . Wiechen, K. (2005). Loss of Gelsolin expression in human ovarian carcinomas. *European Journal of Cancer, 41*(3), 461-469. doi:10.1016/j.ejca.2004.10.025

Pasterkamp, R. J., & Giger, R. J. (2009). Semaphorin function in neural plasticity and disease. *Curr Opin Neurobiol, 19*(3), 263-274. doi:10.1016/j.conb.2009.06.001

Patel, R. R., Khom, S., Steinman, M. Q., Varodayan, F. P., Kiosses, W. B., Hedges, D. M., . . . Roberto, M. (2019). IL-1 beta expression is increased and regulates GABA transmission following chronic ethanol in mouse central amygdala. *Brain Behavior and Immunity, 75*, 208-219. doi:10.1016/j.bbi.2018.10.009

Pineiro, M., Andres, M., Iturralde, M., Carmona, S., Hirvonen, J., Pyorala, S., . . . Alava, M. A. (2004). ITIH4 (inter-alpha-trypsin inhibitor heavy chain 4) is a new acute-phase protein isolated from cattle during experimental infection. *Infection and Immunity, 72*(7), 3777-3782. doi:10.1128/Iai.72.7.3777-3782.2004

Rossignol, P., Ho-Tin-Noe, B., Vranckx, R., Bouton, M. C., Meilhac, O., Lijnen, H. R., . . . Angles-Cano, E. (2004). Protease nexin-1 inhibits plasminogen activation-induced apoptosis of adherent cells. *Journal of Biological Chemistry, 279*(11), 10346-10356. doi:10.1074/jbc.M310964200

Salier, J. P., Rouet, P., Raguenez, G., & Daveau, M. (1996). The inter-alpha-inhibitor family: From structure to regulation. *Biochemical Journal, 315*, 1-9. doi:DOI 10.1042/bj3150001

Sarrazin, S., Lamanna, W. C., & Esko, J. D. (2011). Heparan sulfate proteoglycans. *Cold Spring Harbor perspectives in biology, 3*(7), a004952. doi:10.1101/cshperspect.a004952

Smirnova, T. A., Viskin, A., Hoskova, M., Habartova, L., Setnicka, V., Cejnar, P., & Kuckova, S. (2021). Comparison of proteomic approaches used for the detection of potential biomarkers of Alzheimer's disease in blood plasma. *Journal of Separation Science, 44*(22), 4132-4140. doi:10.1002/jssc.202100468

Tasli, N. G., Cimen, F. K., Karakurt, Y., Ucak, T., Mammadov, R., Suleyman, B., . . . Suleyman, H. (2018). Protective effects of Rutin against methanol induced acute toxic optic neuropathy: an experimental study. *International Journal of Ophthalmology, 11*(5), 780-785. doi:10.18240/ijo.2018.05.10

Yurina, N. V., Ageeva, T. A., Goryachkin, A. M., Varaksin, N. A., Ryabicheva, T. G., Ostanin, A. A., . . . Purtov, A. V. (2021). Effects of Recombinant Angiogenin on Collagen Fiber Formation and Angiogenesis in the Dermis of Wistar Rats. *Clinical Cosmetic and Investigational Dermatology, 14*, 187-196. doi:10.2147/ccid.S294825
