# Supplementary material for: Acute and chronic blood serum proteome changes in patients with methanol poisoning
Source: Sci Rep. 2022 Dec 9;12:21379. doi: 10.1038/s41598-022-25492-9 (PMC9734099; doi:10.1038/s41598-022-25492-9)
Supplement: Supplementary file 11 — Supplementary Information 11. [file 41598_2022_25492_MOESM11_ESM.pdf]

Identification of proteins with significant changes in concentrations between S and C groups

Note: proteins mentioned later in underlined style were detected in the full set of proteins with at least 50% occurrence in any group of M, S, and C (590 proteins). A significant increase or decrease, if not more specified, is related to the first set of the dual set comparison, i.e., M for M vs. S, S for S vs. C, etc. For triple comparison sets, a decrease or increase is related to M, i.e., for M vs. S ∩ M vs. C ∩ S vs. C or M vs. S ∩ M vs. C ∩ M vs. SC. A probable increase or decrease means $0.05 \leq$ q-value < 0.15.

For the coagulation cascade, **coagulation factor X** (increased in S vs. C, M vs. C, M vs. SC) is synthesized in the liver and requires vitamin K. It is activated by hydrolysis into coagulation factor X_a and coagulation factor VII (increased). Therefore, it is the first member of the thrombin pathway. It acts by cleaving prothrombin to the active thrombin. Coagulation factor X_a is inactivated by a protein Z-dependent protease inhibitor (increased), a serine protease inhibitor (serpin). **Heparin cofactor 2** (increased in M vs. C, S vs. C) is a naturally occurring anticoagulant and member of the serpin family. It is uniquely stimulated by the proteoglycan dermatan sulfate (increased in M vs. C, S vs. C). Thrombin is the only coagulation enzyme inhibited by heparin cofactor 2. **Coagulation factor IX** (increased in S vs. C, decreased in M vs. S, probably decreased in M_{pair} vs. S_{pair}) is a proenzyme form of coagulation factor IX_a. It is a substance required for normal blood coagulation. **Vitamin K-dependent protein C** (increased) is the key component of the system, activated by thrombin bound to the membrane protein thrombomodulin. The endothelial protein C receptor further stimulates the protein C activation, which inhibits coagulation by degrading $FVIII_a$ and FV_a coagulation factors (Walker & Fay, 1992). **Vitronectin** (increased in M vs. C, S vs. C, M vs. SC) is a multifunctional glycoprotein that binds glycosaminoglycans, collagen, plasminogen, and the urokinase receptor. It also stabilizes the conformation of plasminogen activation inhibitor-1 (Kamikubo, Neels, & Degryse, 2009). In addition, vitronectin is also involved in the coagulation cascade by binding to heparin and the thrombin-antithrombin III complexes, implicating its participation in the immune response and the regulation of clot formation (Schvartz, Seger, & Shaltiel, 1999).

Regarding other immune and inflammation processes, **complement C1s subcomponent** (increased) is a serine protease that reacts with Complement components C1q and C1r (increased) to form C1 - the first component of the classical pathway of the complement system. C1r activates C1s (increased in S vs. C, probably increased in M vs. S.) so that it can, in turn, activate C2 (increased in M vs. C, probably increased in M vs. SC) and C4. 11 proteins involving complement C1s subcomponent were found that were upregulated in young diabetic rats (Van Kirk et al., 2011). They also demonstrated progressively increasing retinal expression in aged rats. **Plasma protease C1 inhibitor** (increased) controls activation of the C1 complex and may play a key role in the regulation of important physiological pathways, including complement activation, blood coagulation, fibrinolysis, and the generation of kinins. It also acts as a very efficient inhibitor of coagulation factor XII and inhibits chymotrypsin and kallikrein. **Serum amyloid P-component** (increased in M vs. C, S vs. C) and **mannan-binding lectin serine protease 1** (increased in M vs. C, S vs. C) also contribute to the bacterial infection response. Serum amyloid is a component of the humoral arm of innate immunity involved in resistance to bacterial infection and regulation of tissue

remodeling (Doni et al., 2021). Mannan-binding lectin initiates the lectin pathway of complement activation upon binding to microbial carbohydrates (Schwaeble, Dahl, Thiel, Stover, & Jensenius, 2002).

For vitamin A metabolism and lipid transport, **apolipoprotein M** (**ApoM**, increased in M vs. C, S vs. C, M vs. SC) is expressed in the liver and kidneys and is not found free in plasma but is predominantly associated with HDL. It facilitates the formation of pre-beta-HDL and enhances the atheroprotective effects exerted by HDL. Moreover, in patients with diabetes, the levels of plasma apoM may decrease, whereas the augmentation of ApoM decreases plasma glucose levels and magnifies insulin secretion (Ren, Tang, Jiang, Tan, & Yi, 2015).

References

Doni, A., Parente, R., Laface, I., Magrini, E., Cunha, C., Colombo, F. S., . . . Mantovani, A. (2021). Serum amyloid P component is an essential element of resistance against Aspergillus fumigatus. *Nat Commun, 12*(1), 3739. doi:10.1038/s41467-021-24021-y

Kamikubo, Y., Neels, J. G., & Degryse, B. (2009). Vitronectin inhibits plasminogen activator inhibitor-1-induced signalling and chemotaxis by blocking plasminogen activator inhibitor-1 binding to the low-density lipoprotein receptor-related protein. *Int J Biochem Cell Biol, 41*(3), 578-585. doi:10.1016/j.biocel.2008.07.006

Ren, K., Tang, Z. L., Jiang, Y., Tan, Y. M., & Yi, G. H. (2015). Apolipoprotein M. *Clinica Chimica Acta, 446*, 21-29. doi:10.1016/j.cca.2015.03.038

Schvartz, I., Seger, D., & Shaltiel, S. (1999). Vitronectin. *Int J Biochem Cell Biol, 31*(5), 539-544. doi:10.1016/s1357-2725(99)00005-9

Schwaeble, W., Dahl, M. R., Thiel, S., Stover, C., & Jensenius, J. C. (2002). The mannan-binding lectin-associated serine proteases (MASPs) and MAp19: Four components of the lectin pathway activation complex encoded by two genes. *Immunobiology, 205*(4-5), 455-466. doi:10.1078/0171-2985-00146

Van Kirk, C. A., VanGuilder, H. D., Young, M., Farley, J. A., Sonntag, W. E., & Freeman, W. M. (2011). Age-related alterations in retinal neurovascular and inflammatory transcripts. *Molecular Vision, 17*(142), 1261-1274.

Walker, F. J., & Fay, P. J. (1992). REGULATION OF BLOOD-COAGULATION BY THE PROTEIN-C SYSTEM. *Faseb Journal, 6*(8), 2561-2567. doi:10.1096/fasebj.6.8.1317308
